# Genome-wide association study identifies *RNF123* locus as associated with chronic widespread musculoskeletal pain

**DOI:** 10.1136/annrheumdis-2020-219624

**Published:** 2021-04-29

**Authors:** Md Shafiqur Rahman, Bendik S Winsvold, Sergio O Chavez Chavez, Sigrid Børte, Yakov A Tsepilov, Sodbo Zh Sharapov, HUNT All-In Pain, Yurii S Aulchenko, Knut Hagen, Egil A Fors, Kristian Hveem, John Anker Zwart, Joyce B van Meurs, Maxim B Freidin, Frances MK Williams

**Affiliations:** 1 Department of Twin Research and Genetic Epidemiology, School of Life Course Sciences, King's College London, London, UK; 2 Department of Research, Innovation and Education, Division of Clinical Neuroscience, Oslo University Hospital, Oslo, Norway; 3 Department of Neurology, Oslo universitetssykehus Ullevål, Oslo, Norway; 4 K. G. Jebsen Center for Genetic Epidemiology, Department of Public Health and Nursing, Faculty of Medicine and Health Sciences, Norwegian University of Science and Technology, Trondheim, Norway; 5 Department of Internal Medicine, Erasmus Medical Center, Rotterdam, Zuid-Holland, The Netherlands; 6 Research and Communication Unit for Musculoskeletal Health (FORMI), Department of Research, Innovation and Education, Division of Clinical Neuroscience, Oslo University Hospital, Oslo, Norway; 7 Institute of Clinical Medicine, Faculty of Medicine, University of Oslo, Oslo, Norway; 8 Laboratory of Theoretical and Applied Functional Genomics, Novosibirsk State University, Novosibirsk, 630090, Novosibirskaâ, Russia; 9 PolyOmica, ‘s-Hertogenbosch, PA, The Netherlands; 10 Laboratory of Recombination and Segregation Analysis, Institute of Cytology and Genetics, 10 Lavrentiev Avenue, Novosibirsk, 630090, Russia; 11 Department of Neuromedicine and Movement Science, Faculty of Medicine and Health Sciences, Norwegian University of Science and Technology, Trondheim, Norway; 12 Clinical Research Unit Central Norway, St Olavs University Hospital, Trondheim, Norway; 13 Department of Public Health and Nursing, Faculty of Medicine and Health Sciences, Norwegian University of Science and Technology, Trondheim, Norway; 14 HUNT Research Center, Department of Public Health and Nursing, Faculty of Medicine and Health Sciences, Norwegian University of Science and Technology, Trondheim, Norway

**Keywords:** fibromyalgia, polymorphism, genetic, epidemiology

## Abstract

**Background and objectives:**

Chronic widespread musculoskeletal pain (CWP) is a symptom of fibromyalgia and a complex trait with poorly understood pathogenesis. CWP is heritable (48%–54%), but its genetic architecture is unknown and candidate gene studies have produced inconsistent results. We conducted a genome-wide association study to get insight into the genetic background of CWP.

**Methods:**

Northern Europeans from UK Biobank comprising 6914 cases reporting pain all over the body lasting >3 months and 242 929 controls were studied. Replication of three independent genome-wide significant single nucleotide polymorphisms was attempted in six independent European cohorts (n=43 080; cases=14 177). Genetic correlations with risk factors, tissue specificity and colocalisation were examined.

**Results:**

Three genome-wide significant loci were identified (*rs1491985, rs10490825, rs165599*) residing within the genes *Ring Finger Protein 123* (*RNF123*), *ATPase secretory pathway Ca* ^*2+*^ *transporting 1* (*ATP2C1*) and *catechol-O-methyltransferase* (*COMT*). The *RNF123* locus was replicated (meta-analysis p=0.0002), the *ATP2C1* locus showed suggestive association (p=0.0227) and the *COMT* locus was not replicated. Partial genetic correlation between CWP and depressive symptoms, body mass index, age of first birth and years of schooling were identified. Tissue specificity and colocalisation analysis highlight the relevance of skeletal muscle in CWP.

**Conclusions:**

We report a novel association of *RNF123* locus and a suggestive association of *ATP2C1* locus with CWP. Both loci are consistent with a role of calcium regulation in CWP. The association with *COMT*, one of the most studied genes in chronic pain field, was not confirmed in the replication analysis.

**Key messages**

**What is already known about this subject?**

- Chronic widespread musculoskeletal pain (CWP) is a primary diagnostic feature of fibromyalgia.
- CWP is moderately heritable, but precise genes involved in the pathogenesis of CWP are yet to be identified.

**What does this study add?**

- This is the largest genetic study conducted on CWP to date and identified novel genetic risk loci (*Ring Finger Protein 123* and *ATPase secretory pathway Ca* ^*2+*^ *transporting 1*).
- The genetic signal points to peripheral pain mechanisms in CWP, and shows genetic correlation with other traits, including body mass index and depression.

**How might this impact on clinical practice or future developments?**

- The findings add to aetiological basis of CWP.

## Introduction

Chronic widespread musculoskeletal pain (CWP) is a common complex trait influenced by genetic and environmental factors, most of which have yet to be determined.^1^ CWP and fibromyalgia syndrome are sometimes used interchangeably, although the latter is generally more severe and includes other features such as sleep disturbance, fatigue and depression.^2^ It is thought to represent a subgroup at the more severe end of the spectrum of CWP.^3^ The prevalence of CWP is 10.6% in the world population and 14.2% in the UK population.^4 5^ It is associated with high societal cost.^6^ CWP is responsible for excess mortality,^7^ which is thought to be attributable to cardiovascular disease, respiratory disease and cancer. Females are more affected by CWP than males,^4^ and the prevalence rises with age.^5^ In addition to age and sex, a number of exposures have been proposed as risk factors for CWP,^8 9^ but only increased body mass index (BMI) has been consistently reported across studies, including longitudinal studies.^10–12^

Broad-sense heritability estimates for CWP range between 48% and 54%, indicating a substantial genetic contribution.^13^ To date, the candidate gene approach has been extensively applied to identify genetic factors in CWP,^14^ but few agnostic studies have been published.^15^ The only genome-wide association study (GWAS) meta-analysis combining 14 studies identified a locus lying on chromosome 5 intergenic to *CCT5* and *FAM173B*.^15^ *CCT5* has previously been implicated in neuropathy^16^ and there is increasing evidence that small fibre neuropathy underlies a subset of fibromyalgia.^17^

Genetic factors are known to be shared by chronic pain conditions.^18 19^ One of the most extensively studied chronic pain-associated genes encodes *catechol-O-methyltransferase* (*COMT*), an enzyme which regulates the production of catecholamines that act as neurotransmitters in the central nervous system (CNS) pain tract. A non-synonymous change of A to G encoding a valine (Val) to methionine (Met) substitution at codon 158 (*Val158Met*; *rs4680*) reduces the enzymatic activity of *COMT*. This single nucleotide polymorphism (SNP) has been reported to be associated with CWP in a small study of 122 participants,^20^ but a subsequent association study of 3017 participants did not confirm earlier findings.^21^ An inconclusive role of *COMT* was observed for temporomandibular disorders (TMD) as well.^22 23^ Further investigation is required to identify genetic variants underlying CWP, which will shed light on the pathophysiological mechanisms underlying the development of chronic pain and may reveal therapeutic targets.

## Materials and methods

**Figure 1:**
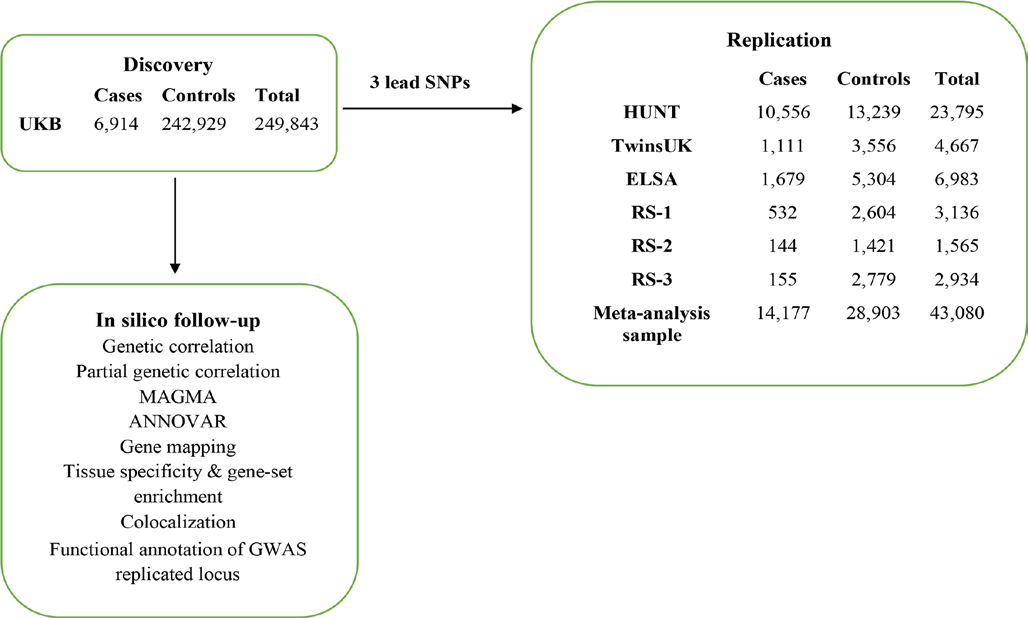
Overview of study design.

An overview of study design is presented in figure 1.

### Participant selection

For the discovery analysis, we performed a GWAS of CWP using UK Biobank (UKB) comprising 249 843 participants of European descent (6914 CWP cases and 242 929 controls). Independent SNPs passing a threshold p<5.0E-08 were submitted for replication in 43 080 individuals of European ancestry (14 177 CWP cases and 28 903 controls) from six independent cohorts originating in the UK (TwinsUK and The English Longitudinal Study of Ageing (ELSA)), the Netherlands (The Rotterdam Study 1, 2 and 3 (RS-1, RS-2 and RS-3)) and Norway (The Nord-Trøndelag Health Survey (HUNT)). The UKB dataset was used under project #18219. Description of each study cohort is presented in online supplemental text.

10.1136/annrheumdis-2020-219624.supp1 Supplementary data

### Phenotype

In UKB, CWP cases were defined by combining self-reported diagnosis of pain all over the body lasting for >3 months; simultaneous pain in the knee, shoulder, hip and back lasting 3+ months and fibromyalgia. Controls comprised those who reported no pain in the last month or reported pain all over the body in the previous month that did not last for 3 months or reported only ≥3 months of non-musculoskeletal pain (headache, facial and abdominal pain). Those reporting a self-reported diagnosis of rheumatoid arthritis, polymyalgia rheumatica, arthritis not otherwise specified, systemic lupus erythematosus, ankylosing spondylitis and myopathy were excluded from the study (online supplemental figure S1). Further phenotype details for UKB and replication cohorts are provided in online supplemental text.

### Genotyping and imputation

Genotyping and imputation methods across cohorts are summarised in online supplemental table S1 (online supplemental text).

### Statistical analysis and in silico follow-up

The details of statistical analysis, and in silico follow-up are described in online supplemental text. In brief, GWAS in the discovery sample was performed using linear mixed-effects model implemented in BOLT-LMM (V.2.3.2).^24^ An additive genetic model for SNP effect on CWP was adjusted for age, sex, genotyping platform and the first 10 genetic principal components provided by UKB. A sensitivity GWAS (controls: 223 606 and CWP cases: 6914) was performed excluding participants with chronic non-musculoskeletal pain such as headache, facial and abdominal pain from the controls. Independent SNPs at GWAS significant loci were identified using Conditional and Joint^25^ analysis and submitted for replication. Independent SNPs across all replication cohorts were meta-analysed using fixed-effects model with both sample size, and inverse-variance weighting implemented in METAL.^26^ SNP heritability was estimated using BOLT-REML^24^ and converted to liability scale. Linkage disequilibrium score regression (LDSR)^27^ was used to estimate inflation in test statistics and genetic correlations. We also estimated partial genetic correlations.^28^ We used Functional Mapping and Annotation (FUMA) webtool^29^ for the annotation of functional consequences of CWP-associated SNPs, gene mapping, tissue specificity and gene-set enrichment. Differential expression of replicated independent SNP was assessed using the GTEx V.8 tissues.^30^ Colocalisation of GWAS-independent SNPs in human skeletal muscle and dorsal root ganglion (DRG) tissues was assessed using publicly available data.^30 31^ Functional annotation of GWAS-replicated locus was performed using Open Targets Platform.^32^

## Results

Details of the discovery and replication cohorts are presented in table 1. Cases were enriched for females compared with controls in all cohorts (p<0.001) and were on average older in the discovery, and in three replication cohorts (p<0.05). In all cohorts, BMI was significantly higher in cases than controls (p<0.0001) except for RS-3 where a similar but non-significant trend was observed (p=0.0827).

**Table 1:** Sample characteristics stratified by case/control status for discovery and replication cohorts

	Cases	Controls	P value
**Discovery cohort (UK Biobank**)			
Female	4470 (64.7%)	128 599 (47.1%)	<0.0001
Male	2444 (35.3%)	114 330 (52.9%)	
Age (mean±SD)	57.8±7.45	57.0±8.09	<0.0001
BMI (mean±SD)	30.02±5.97	26.83±4.40	<0.0001
**Replication cohorts**			
**TwinsUK**			
Female	1041 (93.7%)	3116 (87.6%)	<0.0001
Male	70 (6.3%)	440 (12.4%)	
Age (mean±SD)	54.78±10.48	50.12±13.21	<0.0001
BMI (mean±SD)	27.39±5.11	25.74±4.57	<0.0001
**HUNT**			
Female	6315	5836	<0.0001
Male	4241	7403	
Age (mean±SD)	55.95±9.48	54.82±10.31	<0.0001
BMI (mean±SD)	27.37±4.33	26.52±3.88	<0.0001
**ELSA**			
Female	1090 (64.9%)	2660 (50.2%)	<0.001
Male	589 (35.1%)	2644 (49.8%)	
Age (mean±SD)	68.10±9.49	66.55±9.98	<0.0001
BMI (mean±SD)	28.60±4.98	27.08±4.22	<0.0001
**RS-1**			
Female	422	1323	<0.0001
Male	110	1281	
Age (mean±SD)	64.49±5.30	64.60±5.24	0.6660
BMI (mean±SD)	26.98±3.91	26.14±3.54	<0.0001
**RS-2**			
Female	106	745	<0.0001
Male	38	676	
Age (mean±SD)	61.59±4.59	61.93±4.72	0.2651
BMI (mean±SD)	28.54±4.73	27.77±3.91	0.0363
**RS-3**			
Female	128	1516	<0.0001
Male	27	1263	
Age (mean±SD)	56.28±5.77	56.32±5.46	0.0348
BMI (mean±SD)	28.54±4.86)	27.71±4.62	0.0827

BMI, body mass index; ELSA, The English Longitudinal Study of Ageing; HUNT, The Nord-Trøndelag Health Survey; RS-1, RS-2 and RS-3, The Rotterdam Study 1, 2 and 3; SD, Standard deviation.

### Discovery genome-wide association study

**Figure 2:**
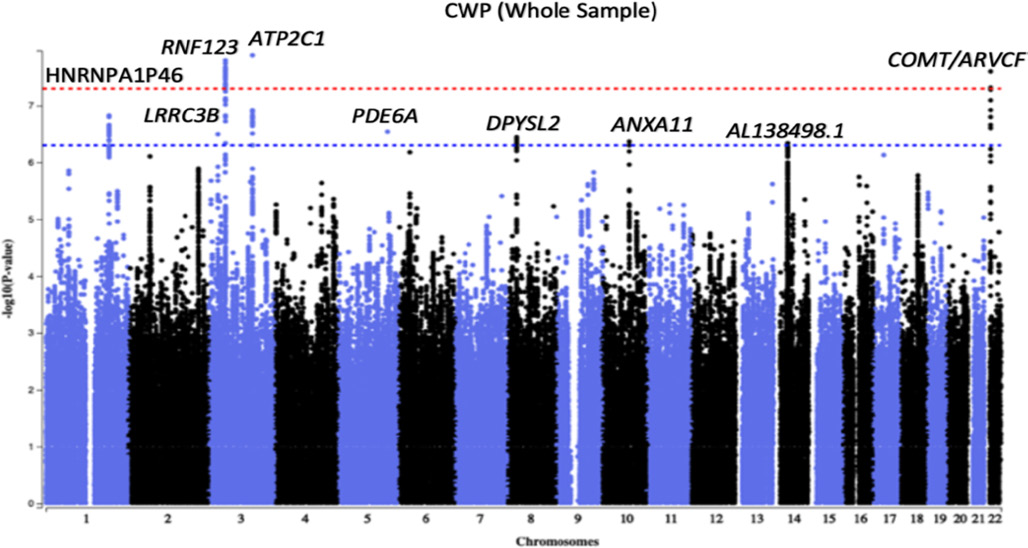
Manhattan plot of a genome-wide association analysis of chronic widespread musculoskeletal pain (CWP). Each circle in the plot represents a single nucleotide polymorphism (SNP), which was positioned following genomic build GRCh37. The y-axis shows the corresponding –log10 p values and the x-axis shows chromosome position along with SNPs. The horizontal red dotted line indicates genome-wide significance threshold at p=5.0×10^–8^. The horizontal blue dotted line indicates suggestive genome-wide significance threshold at p=5.0×10^–7^. Gene labels represent nearest genes to independent SNPs located at loci associated with p<5.0×10^–7^.

**Figure 3:**
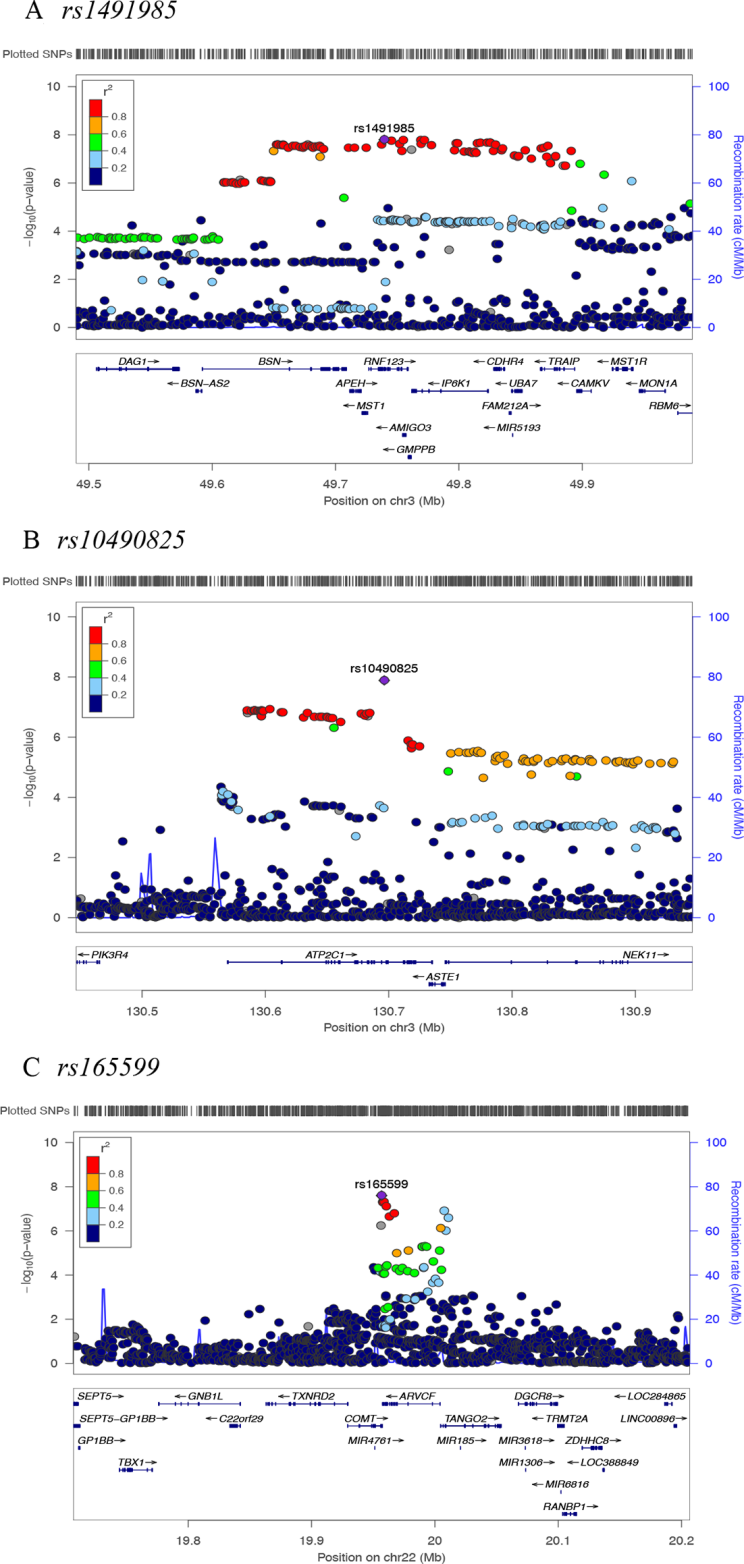
Regional plots for three independent chronic widespread musculoskeletal pain associated single nucleotide polymorphisms (SNPs). Independent SNPs are coloured in purple. Other coloured circles indicate pairwise linkage disequilibrium (LD). The strength of LD (r^2^) presented in the upper left corner of each plot.

Three genomic loci tagged by *rs1491985, rs10490825* and *rs165599* passed genome-wide significance threshold of p<5E-08 (figure 2). Observed inflation in test statistics (λ_GC_=1.146, online supplemental figure S2) was due to polygenicity (LDSR intercept=1.002±0.0085, LDSR ratio=0.0118±0.0497) rather than population stratification. SNP heritability of CWP was 0.05±0.003 on the observed scale, and 0.33±0.0004 on the liability scale meaning that the observed SNPs explain approximately 33% of the variance in CWP risk. Independent SNPs were located in the gene *Ring Finger Protein 123* (*RNF123*) (chromosome 3, *rs1491985,* intronic variant, p=1.60E-08), *ATPase secretory pathway Ca* ^*2+*^ *transporting 1* (*ATP2C1*) (chromosome 3, *rs10490825,* intronic variant, p=1.30E-08) and *COMT* (chromosome 22, rs165599, 3’-untranslated region (3’-UTR) variant, p=2.50E-08), respectively (figure 3A–C; online supplemental table S2). Six additional loci near or within genes *HNRNPA1P46*, *LRRC3B*, *PDE6A*, *DPYSL2*, *ANXA11* and *AL138498*.1 were identified at suggestive GWAS threshold of p<5E-07. Sensitivity GWAS excluding participants with chronic non-musculoskeletal pain provided similar findings except that *COMT* locus now became suggestively significant (p=5.3E-08) (online supplemental figure S3).

### Replication results and meta-analysis

**Figure 4:**
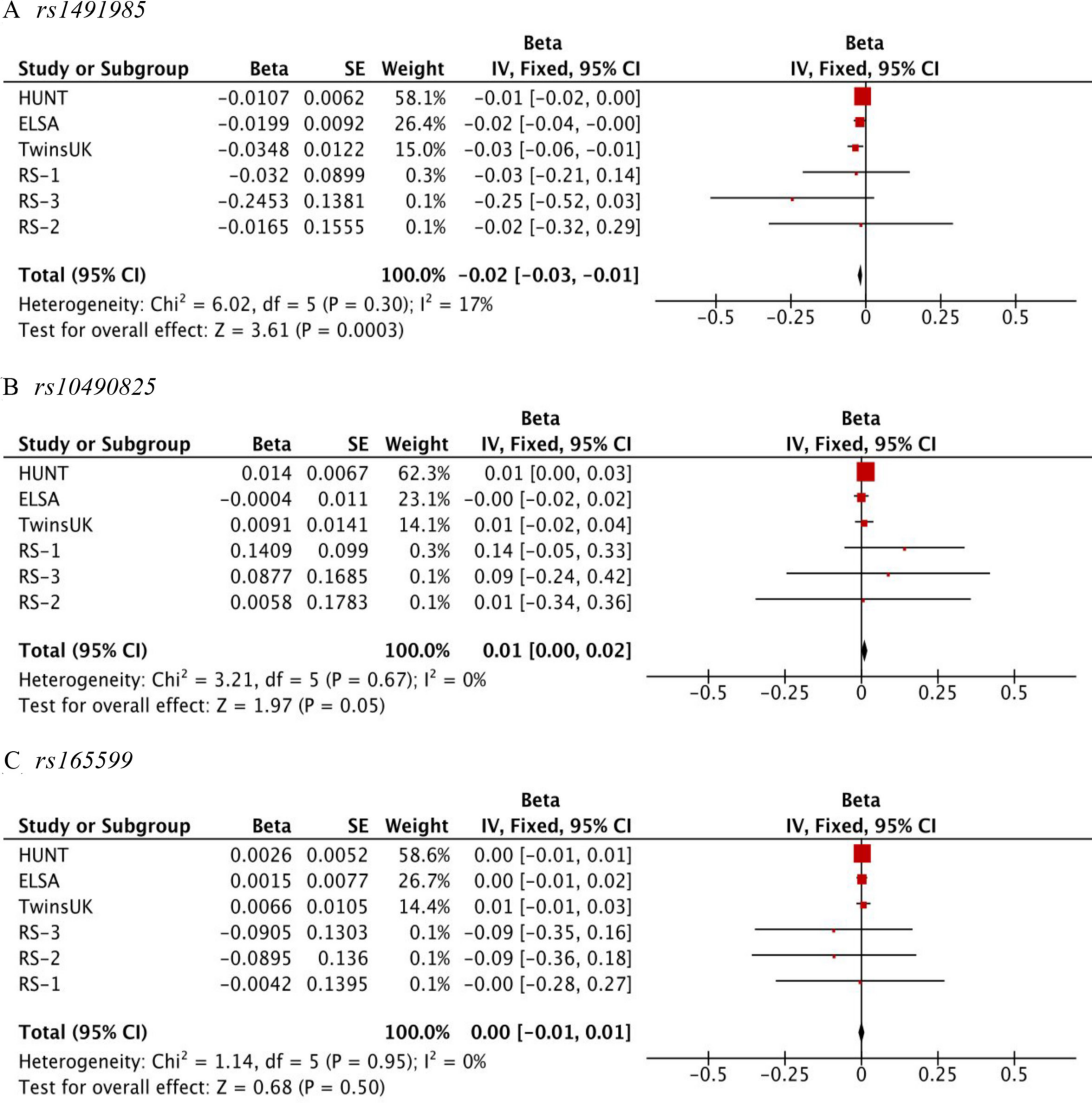
Forest plot for the association of (A) *rs1491985*, (B) *rs10490825,* and (C) *rs165599* with chronic widespread musculoskeletal pain. X-axis shows effect size measures are presented as beta value. The red square with horizontal black line represents the cohort-specific effect with a corresponding CI for the single nucleotide polymorphism (SNP) of interest. Size of the square indicates the weight of the study and reflects sample size. The vertical black line indicates ‘line of no effect’. Overall effect is presented as a black diamond. Test statistics for each cohort, meta-analysis and heterogeneity are available on the left-hand side. The *rs1491985* and *rs10490825* were not present in The English Longitudinal Study of Ageing (ELSA); therefore *rs9870858* and *rs1732984*8 were used as proxy SNPs, respectively (online supplemental text).

Results are presented in online supplemental table S3, with meta-analysis of the six replication samples as shown in figure 4 (online supplemental tables S4, S5). Given the significance threshold for replication: 0.05/3=0.017, association between CWP and *rs1491985* was considered replicated (sample-size based p=0.0002; standard-error based p=0.0003). *Rs10490825* showed suggestive association with CWP (sample-size based p=0.0227; standard-error based p=0.0490) and demonstrated a consistent direction of effect in five of the six replication samples. *Rs165599* did not replicate (sample-size based p=0.7300; standard-error based p=0.5000) and the direction of effect was not consistent across cohorts: in three cohorts, allele A was protective, while in the other three it was the risk allele. None of the three SNPs displayed statistically significant heterogeneity in the replication cohorts.

### CWP shares genetic components with BMI, depression, age at first birth and years of schooling

Two hundred and nine traits from LD-hub (online supplemental text) were examined for genetic correlation with CWP. We selected traits for which the absolute value of the correlation coefficient (r_g_) was >0.2, and for which the Bonferroni-corrected p was <0.01/209=4.78E-05. Twenty-three traits fulfilled these criteria (online supplemental figure S4). The highest positive genetic correlation was observed for depressive symptoms (r_g_=0.65) and the highest negative correlation was observed for college completion (r_g_=−0.61). Many of the 23 genetically correlated traits were correlated with each other raising concerns about their independency of correlations with CWP. We therefore calculated partial genetic correlations conditionally independent of each other. Using hierarchical clustering of genetic correlations we identified seven clusters (online supplemental figure S5A), with seven traits selected to represent each cluster (BMI, triglycerides, depressive symptoms, coronary artery disease, smoking, age of first birth and years of schooling) to quantify partial genetic correlation with CWP. We found depressive symptoms (r_g_=0.59), BMI (r_g_=0.20), age of first birth (r_g_=−0.26) and years of schooling (r_g_=−0.17) independently correlated with CWP (online supplemental figure S5B and table S6).

### Tissue-specific expression of CWP mapped gene sets

**Figure 5:**
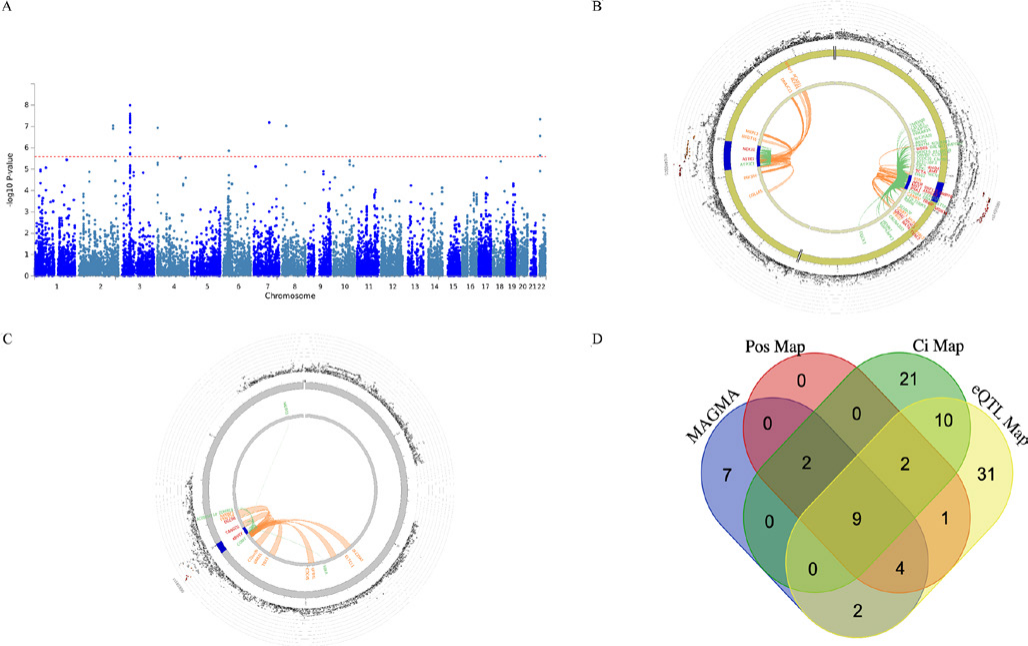
(A) Manhattan plot of the genome-wide gene-based association analysis, (B) & (C) The circus plot displaying chromatin interactions (Ci) and expression quantitative trait loci (eQTLs) on chromosomes 3 and chromosomes 22, respectively, (D) Venn diagram showing overlap of genes implicated by genome-wide gene-based analysis implemented in MAGMA, positional mapping (Pos Map), chromatin interaction mapping (Ci Map), and expression quantitative trait locus mapping (eQTL Map). (A) The y-axis shows the ─log10 transformed two-tailed p-value of each gene from a linear model and the chromosomal position on the x-axis. The red dotted line indicates the Bonferroni-corrected threshold for genome-wide significance of the gene-based test. (B, C) The most outer layer of the circus plot displaying Manhattan plot with –log_10_ p-values for chronic widespread musculoskeletal pain associated independent single nucleotide polymorphisms (SNPs). Each SNP is presented with rsID. Linkage disequilibrium (LD) relationship between independent SNPs at the locus and their proxies are indicated with red (r^2^ > 0.8) and orange (r^2^ > 0.6). Grey SNPs indicate minimal LD with r^2^ ≤0.20. The outer circle represents chromosome with genomic risk loci are highlighted in blue. Either Ci- or eQTL mapped genes are displayed on the inner circle. Ci- and eQTL mapped genes are presented in orange or green color, respectively. Genes mapped with both approaches are colored red.

**Figure 6:**
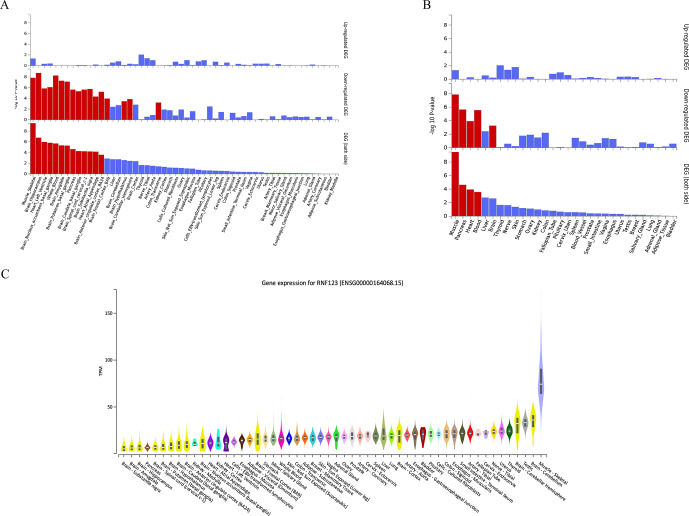
(A) Differentially expressed gene (DEG) plots for chronic widespread musculoskeletal pain (CWP) in 54 tissue types from GTEX v8, (B) DEG plots for CWP in 30 general tissue types from GTEX v8 and (C) Differential expression of *RNF123* gene across tissue types from GTEX v8. (A, B) In both plots, the y-axis represents the ─log10 transformed two-tailed p value of the hypergeometric test. Significantly enriched DEG sets (Bonferroni-corrected p value <0.05) are highlighted in red. (C) Y-axis represents transcripts per million (TPM) and x-axis represents the GTEx (V.8) tissues. The figure was adapted from GTEx portal (https://www.gtexportal.org/home/gene/ENSG00000164068).

The results of functional consequences of GWAS-independent SNPs and their proxies are presented in online supplemental figure S6 (online supplemental text). Four different gene mapping strategies were implemented in FUMA (genome-wide gene-based association analysis, positional, expression quantitative trait locus (eQTL) and chromatin interaction mapping) linking annotated SNPs to 89 genes of which *MST1, GMPPB, APEH, RNF123, ARVCF, AMIGO3, IP6K1, TANGO2* and *TRAIP* were identified using all four methods (figure 5A–D).^33^ Mapped genes were investigated for tissue-specific gene expression and gene-set enrichment. In 54 specific GTEx tissues types, differentially expressed gene sets enriched for skeletal muscle, several brain tissues, heart, whole blood, pancreas and transverse colon (figure 6A, online supplemental table S7). In 30 general GTEx tissue types, differentially expressed gene sets enriched for skeletal muscle, pancreas, heart, blood and brain (figure 6B, online supplemental table S8). In both sets of GTEx tissues, overall enrichment for differentially expressed gene sets containing *RNF123* and *ATP2C1* genes were stronger for skeletal muscle than other tissues. *RNF123* was found to be highly expressed in skeletal muscle compared with other tissue types (figure 6C). None of the hallmark gene sets available in the molecular signature database was identified in the analysis.

### Putative causal genes in *RNF123* locus

Colocalisation analysis identified a 93% probability of shared eQTL variant *rs6809879,* which controls *Cadherin Related Family Member 4* (*CDHR4*) expression in the skeletal muscle and CWP association signal near the *RNF123* locus (online supplemental table S9, online supplemental figure S7A). Additionally, significant colocalisation was found for *rs13093525*, which controls *APEH* expression in DRG at exon level (72% probability of shared variant with *RNF123* locus). Both *rs6809879* and *rs13093525* were in complete LD with independent SNP *rs1491985* (R^2^=1) (online supplemental table S10, online supplemental figure S7B). No evidence of skeletal muscle or DRG eQTL colocalisation was observed for *ATP2C1* and *COMT* loci. Functional annotation of *RNF123* locus identified nine genes (SLC25A20, *NDUFAF3*, *DAG1*, *HYAL1, GMPPB, TRAIP, RHOA, CACNA2D2* and *IMPDH2*) specific to musculoskeletal system diseases, of which *CACNA2D2*, *NDUFAF3* and *IMPDH2* enriched as druggable targets (online supplemental figure S8).

## Discussion

CWP is a prevalent condition with moderate heritability and serves as a cardinal diagnostic feature of fibromyalgia. Therefore, our findings are of importance for better understanding the genetic basis of fibromyalgia. We report here the largest GWAS of CWP to date using 249 843 participants from the UKB, identifying 3 genome-wide significant loci implicating *RNF123*, *ATP2C1* and *COMT*. The association in *RNF123* was replicated, whereas *ATP2C1* showed a suggestive association, and the *COMT* locus did not replicate in 43 080 individuals from independent cohorts.

*RNF123* gene encodes E3 ubiquitin-protein ligase, has a role in cell cycle progression, metabolism of proteins and innate immunity.^34 35^ This gene is highly expressed in skeletal muscle than other tissues. Recent studies involving UKB samples also associated the locus with musculoskeletal pain.^19 36^ However, it is not clear how *RNF123* may contribute to CWP. Using in silico follow-up, we identified *CDHR4*, *APEH*, *SLC25A20*, *NDUFAF3*, *DAG1*, *HYAL1*, *GMPPB*, *TRAIP*, *RHOA*, *CACNA2D2* and *IMPDH2* genes as putative causal candidates at the locus, of which *CACNA2D2*, *NDUFAF3* and *IMPDH2* can be targeted using known drugs.^37–39^ Notably, *CACNA2D2* encodes the alpha-2/delta subunit of the voltage-dependent calcium channel complex, which is a receptor for gabapentinoids,^40^ used by some in the management of fibromyalgia.^41 42^ Another prioritised gene *CDHR4* belongs to cadherin superfamily has a role in calcium-ion binding to facilitate cadherin-mediated cell-cell interaction.^43 44^

Additionally, the *ATP2C1* locus demonstrated suggestive association in replication (p=0.0227). There was a consistent direction of effect for *ATP2C1* locus in six replication cohorts but not ELSA, where we used a proxy SNP, which had close to zero effect size (beta=−0.0004±0.0110). This is the first study to implicate *ATP2C1* with musculoskeletal pain using an agnostic approach. The *ATP2C1* gene encodes for the ATP-powered magnesium-dependent calcium pump protein hSPCA1, which mediates Golgi uptake of cytosolic Ca(2+) and Mg(2+).^45^ A loss of function mutation in the *ATP2C1* leads to Hailey-Hailey disease (HHD), an autosomal dominant skin condition characterised by blistering and erosion of the epidermis.^46^ Interestingly, HHD may be treated successfully with low-dose naltrexone, an opioid receptor antagonist, which has also been used in the management of fibromyalgia.^47 48^ A recent study showed that naltrexone is capable of restoring calcium homeostasis in natural killer cells of patients with chronic fatigue syndrome.^49^ Additionally, the role of calcium regulation in pain processing is well known.^50–52^ Taken together, our findings suggest a role in the regulation of calcium influencing CWP/fibromyalgia.

*COMT* is one of the most studied genes in human pain.^53^ Almost 30 SNPs and 3 haploblocks of the *COMT* gene have been studied in acute clinical, experimental and chronic pain. *Rs4680* of the *COMT* gene is extensively studied in many pain phenotypes such as pain sensitivity, TMD and fibromyalgia.^54^ Across multiple ethnic populations, *rs4680* was implicated with fibromyalgia.^55^ However, a meta-analysis of 8 case-control studies (589 fibromyalgia cases and 527 controls) did not confirm earlier association.^56^ To date, the largest study that assessed the association between *COMT* haplotypes (*rs4680, rs4818, rs4633* and *rs6269*) and fibromyalgia included 60 367 participants (2713 ICD-9 diagnosed fibromyalgia) and found no association.^57^ They have also been refuted in other European CWP samples^21 58^ and a large candidate gene study of fibromyalgia.^59^ However, we identified *rs165599*, located at 3’-UTR of *COMT*, associated with CWP in the discovery sample but not in the meta-analysis or any of the replication cohorts. This variant is not in LD with previously studied *COMT* SNPs *rs4680, rs4818, rs4633* and *rs6269,* and was found not to be associated with chronic musculoskeletal pain including CWP neither when studied as a single SNP nor as a part of a haploblock.^60–62^ Several explanations of our non-replication of *COMT* locus are possible. First, there was lower power pertaining to overall meta-analysis, which was estimated at 48% based on the effect size observed in the discovery sample (n=249 843), replication sample size (n=43 080) and the number of tests conducted (n=3). Our meta-analysis did have 90% power to detect a relative risk as small as 1.04 but the estimated *COMT* effect was only 1.012 (beta=0.0027±0.004; OR=1.012, 95% CI=0.97 to 1.05). However, our replication sample size was larger than many of the earlier studies that reported the association between *COMT* and CWP.^20 63^ Second, we observed a tendency towards non-significance for the *COMT* locus in the sensitivity GWAS due to the exclusion of participants with non-musculoskeletal pain from the control group suggesting that *COMT* predisposes to chronic pain in general. Finally, genetic factors underlying chronic pain and psychiatric comorbidity (e.g. depression and neuroticism) are known to be shared.^64^ However, previous GWAS on chronic pain,^28 65 66^ depression^67^ and neuroticism^68^ have failed to detect an association with *COMT*. Thus, if there is a role of *COMT* in CWP, it is likely minimal.

Epidemiological studies have consistently reported higher BMI to be associated with an increased risk of CWP.^10 11 69^ Our analysis showed significantly higher BMI in CWP cases compared with controls (p<0.0001) in all cohorts except RS-3. In line with this, we observed a positive genetic overlap between BMI and CWP independent of genetic confounders. Similarly, genetically independent pairwise genetic correlation for depressive symptoms, age of first birth and years of schooling was seen with CWP. These findings indicate the presence of shared molecular pathways underlying these traits.

Functional analysis showed that FUMA mapped genes differentially expressed in skeletal muscle, several areas of the CNS, pancreas, whole blood and heart tissues. These findings suggest the involvement of nervous, musculoskeletal and neuroendocrine systems in CWP. These physiological systems have been implicated in fibromyalgia by previous studies.^70–72^ Evidence suggests that both peripheral and central pain mechanisms influence CWP.^73 74^ We observed overall stronger enrichment for differentially expressed gene sets in skeletal muscle than other GTEx tissues. Also, skeletal muscle and DRG eQTLs colocalise with the *RNF123* locus. These findings suggest a substantial involvement of peripheral pain mechanisms in CWP.

The study has limitations. The case definition of CWP depends on self-report together with exclusion of other conditions with symptoms leading to chronic pain.^75^ A clinical diagnosis of CWP would have been infeasible in a sample this large. Also, we used common SNPs to estimate the heritability of CWP, so the contribution of other variants in the heritability estimated remains unknown. The phenotype definition used in this study to estimate SNP heritability has differed from the Kato *et al* 13 study, where a modulated American College of Rheumatology^76^ criteria based on self-report was used to estimate broad-sense heritability. However, using UKB samples, a study reported the SNP heritability of pain all over the body, regardless of chronicity, on the liability scale was 0.31±0.072.^64^ We found a similar but slightly higher estimate for CWP (0.33±0.0004), suggesting our definition is meaningful and CWP is a trait of high genetic influence. Finally, our findings cannot be generalisable to ancestry other than northern Europeans (online supplemental text).

In summary, this study identified a novel association for CWP in the *RNF123* locus and suggested the role of calcium regulation, by the involvement of the *CDHR4, CACNA2D2* and *ATP2C1* genes. The association of the *COMT* locus with CWP was not replicated, suggesting a small influence, if any. We found evidence that the epidemiological association of BMI and CWP is at least in part genetically mediated. Finally, our results suggest a profound role of peripheral mechanisms in the pathogenesis of CWP.

## Data Availability

Summary statistics from our discovery and sensitivity GWAS was deposited at Zenodo (https://doi.org/10.5281/zenodo.4459546). Other data relevant to the study are included in the article or uploaded as online supplementary information.

## References

[1] Häuser W, Ablin J, Fitzcharles M-A, et al. Fibromyalgia. Nat Rev Dis Primers 2015;1:15022. 10.1038/nrdp.2015.22 27189527

[2] Wolfe F, Clauw DJ, Fitzcharles M-A, et al. 2016 revisions to the 2010/2011 fibromyalgia diagnostic criteria. Semin Arthritis Rheum 2016;46:319–29. 10.1016/j.semarthrit.2016.08.012 27916278

[3] Shipley M. Chronic widespread pain and fibromyalgia syndrome. Medicine 2018;46:252–5. 10.1016/j.mpmed.2018.01.009

[4] Fayaz A, Croft P, Langford RM, et al. Prevalence of chronic pain in the UK: a systematic review and meta-analysis of population studies. BMJ Open 2016;6:e010364. 10.1136/bmjopen-2015-010364 PMC4932255 27324708

[5] Mansfield KE, Sim J, Jordan JL, et al. A systematic review and meta-analysis of the prevalence of chronic widespread pain in the general population. Pain 2016;157:55–64. 10.1097/j.pain.0000000000000314 26270591 PMC4711387

[6] Boonen A, van den Heuvel R, van Tubergen A, et al. Large differences in cost of illness and wellbeing between patients with fibromyalgia, chronic low back pain, or ankylosing spondylitis. Ann Rheum Dis 2005;64:396–402. 10.1136/ard.2003.019711 15271773 PMC1755408

[7] Macfarlane GJ, Barnish MS, Jones GT. Persons with chronic widespread pain experience excess mortality: longitudinal results from UK Biobank and meta-analysis. Ann Rheum Dis 2017;76:1815–22. 10.1136/annrheumdis-2017-211476 28733474

[8] Kvalheim S, Sandven I, Hagen K, et al. Smoking as a risk factor for chronic musculoskeletal complaints is influenced by age. The HUNT study. Pain 2013;154:1073–9. 10.1016/j.pain.2013.03.015 23623251

[9] Kvalheim S, Sandvik L, Winsvold B, et al. Early menarche and chronic widespread musculoskeletal complaints--Results from the HUNT study. Eur J Pain 2016;20:458–64. 10.1002/ejp.747 26132558

[10] Mundal I, Gråwe RW, Bjørngaard JH, et al. Prevalence and long-term predictors of persistent chronic widespread pain in the general population in an 11-year prospective study: the HUNT study. BMC Musculoskelet Disord 2014;15:213. 10.1186/1471-2474-15-213 24951013 PMC4089927

[11] Mundal I, Gråwe RW, Bjørngaard JH, et al. Psychosocial factors and risk of chronic widespread pain: an 11-year follow-up study--the HUNT study. Pain 2014;155:1555–61. 10.1016/j.pain.2014.04.033 24813831

[12] Wright LJ, Schur E, Noonan C, et al. Chronic pain, overweight, and obesity: findings from a community-based twin registry. J Pain 2010;11:628–35. 10.1016/j.jpain.2009.10.004 20338816 PMC2892725

[13] Kato K, Sullivan PF, Evengård B, et al. Importance of genetic influences on chronic widespread pain. Arthritis Rheum 2006;54:1682–6. 10.1002/art.21798 16646040

[14] Kerr JI, Burri A. Genetic and epigenetic epidemiology of chronic widespread pain. J Pain Res 2017;10:2021–9. 10.2147/JPR.S143869 28894385 PMC5584918

[15] Peters MJ, Broer L, Willemen HLDM, et al. Genome-wide association study meta-analysis of chronic widespread pain: evidence for involvement of the 5p15.2 region. Ann Rheum Dis 2013;72:427–36. 10.1136/annrheumdis-2012-201742 22956598 PMC3691951

[16] Bouhouche A, Benomar A, Bouslam N, et al. Mutation in the epsilon subunit of the cytosolic chaperonin-containing t-complex peptide-1 (CCT5) gene causes autosomal recessive mutilating sensory neuropathy with spastic paraplegia. J Med Genet 2006;43:441–3. 10.1136/jmg.2005.039230 16399879 PMC2564519

[17] Lawson VH, Grewal J, Hackshaw KV, et al. Fibromyalgia syndrome and small fiber, early or mild sensory polyneuropathy. Muscle Nerve 2018;58:625–30. 10.1002/mus.26131 29572887 PMC6283273

[18] Vehof J, Zavos HMS, Lachance G, et al. Shared genetic factors underlie chronic pain syndromes. Pain 2014;155:1562–8. 10.1016/j.pain.2014.05.002 24879916

[19] Tsepilov YA, Freidin MB, Shadrina AS. Analysis of genetically independent phenotypes identifies shared genetic factors associated with chronic musculoskeletal pain at different anatomic sites. bioRxiv 2019;810283. 10.1038/s42003-020-1051-9 PMC7316754 32587327

[20] Gürsoy S, Erdal E, Herken H, et al. Significance of catechol-O-methyltransferase gene polymorphism in fibromyalgia syndrome. Rheumatol Int 2003;23:104–7. 10.1007/s00296-002-0260-5 12739038

[21] Hagen K, Pettersen E, Stovner LJ, et al. No association between chronic musculoskeletal complaints and Val158Met polymorphism in the catechol-O-methyltransferase gene. The HUNT study. BMC Musculoskelet Disord 2006;7:40. 10.1186/1471-2474-7-40 16674809 PMC1524765

[22] Diatchenko L, Nackley AG, Slade GD, et al. Catechol-O-methyltransferase gene polymorphisms are associated with multiple pain-evoking stimuli. Pain 2006;125:216–24. 10.1016/j.pain.2006.05.024 16837133

[23] Smith SB, Parisien M, Bair E, et al. Genome-Wide association reveals contribution of MRAS to painful temporomandibular disorder in males. Pain 2019;160:579–91. 10.1097/j.pain.0000000000001438 30431558 PMC6377338

[24] Loh P-R, Tucker G, Bulik-Sullivan BK, et al. Efficient Bayesian mixed-model analysis increases association power in large cohorts. Nat Genet 2015;47:284–90. 10.1038/ng.3190 25642633 PMC4342297

[25] Yang J, Ferreira T, Morris AP, et al. Conditional and joint multiple-SNP analysis of GWAS summary statistics identifies additional variants influencing complex traits. Nat Genet 2012;44:369–75. 10.1038/ng.2213 22426310 PMC3593158

[26] Willer CJ, Li Y, Abecasis GR. Metal: fast and efficient meta-analysis of genomewide association scans. Bioinformatics 2010;26:2190–1. 10.1093/bioinformatics/btq340 20616382 PMC2922887

[27] Bulik-Sullivan BK, Loh P-R, Finucane HK, et al. Ld score regression distinguishes confounding from polygenicity in genome-wide association studies. Nat Genet 2015;47:291–5. 10.1038/ng.3211 25642630 PMC4495769

[28] Freidin MB, Tsepilov YA, Palmer M, et al. Insight into the genetic architecture of back pain and its risk factors from a study of 509,000 individuals. Pain 2019;160:1361–73. 10.1097/j.pain.0000000000001514 30747904 PMC7066867

[29] Watanabe K, Taskesen E, van Bochoven A, et al. Functional mapping and annotation of genetic associations with FUMA. Nat Commun 2017;8:1826. 10.1038/s41467-017-01261-5 29184056 PMC5705698

[30] GTEx Consortium. The GTEx Consortium atlas of genetic regulatory effects across human tissues. Science 2020;369:1318–30. 10.1126/science.aaz1776 32913098 PMC7737656

[31] Parisien M, Khoury S, Chabot-Doré A-J, et al. Effect of human genetic variability on gene expression in dorsal root ganglia and association with pain phenotypes. Cell Rep 2017;19:1940–52. 10.1016/j.celrep.2017.05.018 28564610 PMC5524461

[32] Carvalho-Silva D, Pierleoni A, Pignatelli M, et al. Open targets platform: new developments and updates two years on. Nucleic Acids Res 2019;47:D1056–65. 10.1093/nar/gky1133 30462303 PMC6324073

[33] Heberle H, Meirelles GV, da Silva FR, et al. InteractiVenn: a web-based tool for the analysis of sets through Venn diagrams. BMC Bioinformatics 2015;16:169. 10.1186/s12859-015-0611-3 25994840 PMC4455604

[34] Kamura T, Hara T, Matsumoto M, et al. Cytoplasmic ubiquitin ligase KPC regulates proteolysis of p27(Kip1) at G1 phase. Nat Cell Biol 2004;6:1229–35. 10.1038/ncb1194 15531880

[35] Wang S, Yang Y-K, Chen T, et al. RNF123 has an E3 ligase-independent function in RIG-I-like receptor-mediated antiviral signaling. EMBO Rep 2016;17:1155–68. 10.15252/embr.201541703 27312109 PMC4967948

[36] Johnston KJA, Adams MJ, Nicholl BI, et al. Genome-Wide association study of multisite chronic pain in UK Biobank. PLoS Genet 2019;15:e1008164. 10.1371/journal.pgen.1008164 31194737 PMC6592570

[37] Vinik A, Rosenstock J, Sharma U, et al. Efficacy and safety of mirogabalin (DS-5565) for the treatment of diabetic peripheral neuropathic pain: a randomized, double-blind, placebo- and active comparator-controlled, adaptive proof-of-concept phase 2 study. Diabetes Care 2014;37:3253–61. 10.2337/dc14-1044 25231896

[38] Emami Riedmaier A, Fisel P, Nies AT, et al. Metformin and cancer: from the old medicine cabinet to pharmacological pitfalls and prospects. Trends Pharmacol Sci 2013;34:126–35. 10.1016/j.tips.2012.11.005 23277337

[39] Sanquer S, Maison P, Tomkiewicz C, et al. Expression of inosine monophosphate dehydrogenase type I and type II after mycophenolate mofetil treatment: a 2-year follow-up in kidney transplantation. Clin Pharmacol Ther 2008;83:328–35. 10.1038/sj.clpt.6100300 17713475

[40] Patel R, Dickenson AH. Mechanisms of the gabapentinoids and α 2 δ-1 calcium channel subunit in neuropathic pain. Pharmacol Res Perspect 2016;4:e00205. 10.1002/prp2.205 27069626 PMC4804325

[41] Derry S, Cording M, Wiffen PJ, et al. Pregabalin for pain in fibromyalgia in adults. Cochrane Database Syst Rev 2016;9:Cd011790. 10.1002/14651858.CD011790.pub2 27684492 PMC6457745

[42] Cooper TE, Derry S, Wiffen PJ, et al. Gabapentin for fibromyalgia pain in adults. Cochrane Database Syst Rev 2017;56:CD012188. 10.1002/14651858.CD012188.pub2 PMC6465053 28045473

[43] Sotomayor M, Gaudet R, Corey DP. Sorting out a promiscuous superfamily: towards cadherin connectomics. Trends Cell Biol 2014;24:524–36. 10.1016/j.tcb.2014.03.007 24794279 PMC4294768

[44] Cailliez F, Lavery R. Cadherin mechanics and complexation: the importance of calcium binding. Biophys J 2005;89:3895–903. 10.1529/biophysj.105.067322 16183887 PMC1366956

[45] Micaroni M, Giacchetti G, Plebani R, et al. ATP2C1 gene mutations in Hailey-Hailey disease and possible roles of SPCA1 isoforms in membrane trafficking. Cell Death Dis 2016;7:e2259. 10.1038/cddis.2016.147 27277681 PMC5143377

[46] Sudbrak R, Brown J, Dobson-Stone C, et al. Hailey-Hailey disease is caused by mutations in ATP2C1 encoding a novel Ca(2+) pump. Hum Mol Genet 2000;9:1131–40. 10.1093/hmg/9.7.1131 10767338

[47] Kollman N, Bass J. Generalized familial benign chronic pemphigus (Hailey-Hailey disease) treated successfully with low-dose naltrexone. JAAD Case Rep 2018;4:725–7. 10.1016/j.jdcr.2018.07.002 30167446 PMC6113657

[48] Albers LN, Arbiser JL, Feldman RJ. Treatment of Hailey-Hailey disease with low-dose naltrexone. JAMA Dermatol 2017;153:1018–20. 10.1001/jamadermatol.2017.2446 28768313 PMC5817589

[49] Cabanas H, Muraki K, Staines D, et al. Naltrexone restores impaired transient receptor potential melastatin 3 ion channel function in natural killer cells from myalgic Encephalomyelitis/Chronic fatigue syndrome patients. Front Immunol 2019;10:2545. 10.3389/fimmu.2019.02545 31736966 PMC6834647

[50] Park J, Luo ZD. Calcium channel functions in pain processing. Channels 2010;4:510–7. 10.4161/chan.4.6.12869 21150297 PMC3052250

[51] Bourinet E, Altier C, Hildebrand ME, et al. Calcium-permeable ion channels in pain signaling. Physiol Rev 2014;94:81–140. 10.1152/physrev.00023.2013 24382884

[52] Younger J, Noor N, McCue R, et al. Low-dose naltrexone for the treatment of fibromyalgia: findings of a small, randomized, double-blind, placebo-controlled, counterbalanced, crossover trial assessing daily pain levels. Arthritis Rheum 2013;65:529–38. 10.1002/art.37734 23359310

[53] Mogil JS. Pain genetics: past, present and future. Trends Genet 2012;28:258–66. 10.1016/j.tig.2012.02.004 22464640

[54] edKambur O, Männistö PT. Catechol-O-Methyltransferase and pain. Int Rev Neurobiol 2010;95:227–79. 10.1016/B978-0-12-381326-8.00010-7 21095465

[55] Park D-J, Lee S-S. New insights into the genetics of fibromyalgia. Korean J Intern Med 2017;32:984–95. 10.3904/kjim.2016.207 29056037 PMC5668398

[56] Zhang L, Zhu J, Chen Y, et al. Meta-Analysis reveals a lack of association between a common catechol-O-methyltransferase (COMT) polymorphism val¹⁵⁸met and fibromyalgia. Int J Clin Exp Pathol 2014;7:8489–97. 25674213 PMC4314034

[57] Lee C, Liptan G, Kantorovich S, et al. Association of Catechol-O-methyltransferase single nucleotide polymorphisms, ethnicity, and sex in a large cohort of fibromyalgia patients. BMC Rheumatol 2018;2:38. 10.1186/s41927-018-0045-4 30886988 PMC6390547

[58] Nicholl BI, Holliday KL, Macfarlane GJ, et al. No evidence for a role of the catechol-O-methyltransferase pain sensitivity haplotypes in chronic widespread pain. Ann Rheum Dis 2010;69:2009–12. 10.1136/ard.2009.126086 20570835

[59] Smith SB, Maixner DW, Fillingim RB, et al. Large candidate gene association study reveals genetic risk factors and therapeutic targets for fibromyalgia. Arthritis Rheum 2012;64:584–93. 10.1002/art.33338 21905019 PMC3237946

[60] Vargas-Alarcón G, Fragoso J-M, Cruz-Robles D, et al. Catechol-O-Methyltransferase gene haplotypes in Mexican and Spanish patients with fibromyalgia. Arthritis Res Ther 2007;9:R110. 10.1186/ar2316 17961261 PMC2212567

[61] Hocking LJ, Smith BH, Jones GT, et al. Genetic variation in the beta2-adrenergic receptor but not catecholamine-O-methyltransferase predisposes to chronic pain: results from the 1958 British birth cohort study. Pain 2010;149:143–51. 10.1016/j.pain.2010.01.023 20167428

[62] Diatchenko L, Slade GD, Nackley AG, et al. Genetic basis for individual variations in pain perception and the development of a chronic pain condition. Hum Mol Genet 2005;14:135–43. 10.1093/hmg/ddi013 15537663

[63] Tammimäki A, Männistö PT. Catechol-O-Methyltransferase gene polymorphism and chronic human pain: a systematic review and meta-analysis. Pharmacogenet Genomics 2012;22:673–91. 10.1097/FPC.0b013e3283560c46 22722321

[64] Meng W, Adams MJ, Reel P, et al. Genetic correlations between pain phenotypes and depression and neuroticism. Eur J Hum Genet 2020;28:358–66. 10.1038/s41431-019-0530-2 31659249 PMC7028719

[65] Meng W, Chan BW, Harris C, et al. A genome-wide association study finds genetic variants associated with neck or shoulder pain in UK Biobank. Hum Mol Genet 2020;29:1396–404. 10.1093/hmg/ddaa058 32246137 PMC7254846

[66] Meng W, Adams MJ, Palmer CNA, et al. Genome-wide association study of knee pain identifies associations with GDF5 and COL27A1 in UK Biobank. Commun Biol 2019;2:321. 10.1038/s42003-019-0568-2 31482140 PMC6713725

[67] Howard DM, Adams MJ, Clarke T-K, et al. Genome-Wide meta-analysis of depression identifies 102 independent variants and highlights the importance of the prefrontal brain regions. Nat Neurosci 2019;22:343–52. 10.1038/s41593-018-0326-7 30718901 PMC6522363

[68] Nagel M, Jansen PR, Stringer S, et al. Meta-Analysis of genome-wide association studies for neuroticism in 449,484 individuals identifies novel genetic loci and pathways. Nat Genet 2018;50:920–7. 10.1038/s41588-018-0151-7 29942085

[69] Mork PJ, Vasseljen O, Nilsen TIL. Association between physical exercise, body mass index, and risk of fibromyalgia: longitudinal data from the Norwegian Nord-Trøndelag health study. Arthritis Care Res 2010;62:611–7. 10.1002/acr.20118 20191480

[70] Olsen NJ, Park JH. Skeletal muscle abnormalities in patients with fibromyalgia. Am J Med Sci 1998;315:351–8. 10.1097/00000441-199806000-00003 9638891

[71] Staud R. Autonomic dysfunction in fibromyalgia syndrome: postural orthostatic tachycardia. Curr Rheumatol Rep 2008;10:463–6. 10.1007/s11926-008-0076-8 19007537

[72] Furlan R, Colombo S, Perego F, et al. Abnormalities of cardiovascular neural control and reduced orthostatic tolerance in patients with primary fibromyalgia. J Rheumatol 2005;32:1787–93. 16142879

[73] Sluka KA, Clauw DJ. Neurobiology of fibromyalgia and chronic widespread pain. Neuroscience 2016;338:114–29. 10.1016/j.neuroscience.2016.06.006 27291641 PMC5083139

[74] Staud R. Peripheral pain mechanisms in chronic widespread pain. Best Pract Res Clin Rheumatol 2011;25:155–64. 10.1016/j.berh.2010.01.010 22094192 PMC3220877

[75] Häuser W, Perrot S, Sommer C, et al. Diagnostic confounders of chronic widespread pain: not always fibromyalgia. Pain Rep 2017;2:e598. 10.1097/PR9.0000000000000598 29392213 PMC5741304

[76] Wolfe F, Smythe HA, Yunus MB, et al. The American College of rheumatology 1990 criteria for the classification of fibromyalgia. Report of the multicenter criteria Committee. Arthritis Rheum 1990;33:160–72. 10.1002/art.1780330203 2306288

