# Supplementary material for: Genome-wide association study identifies RNF123 locus as associated with chronic widespread musculoskeletal pain
Source: Ann Rheum Dis. 2021 Apr 29;80(9):1227–35. doi: 10.1136/annrheumdis-2020-219624 (PMC8372387; doi:10.1136/annrheumdis-2020-219624)
Supplement: Supplementary data [file annrheumdis-2020-219624supp001.pdf]

Online Supplemental Material

Genome-wide association study identifies *RNF123* locus as associated with chronic widespread musculoskeletal pain

Md Shafiqur Rahman[1], Bendik S Winsvold[2,3,4], Sergio O Chavez Chavez[11], Sigrid Børte[4,5,6], Yakov A. Tsepilov [12,13,14], Sodbo Zh. Sharapov[12,14], *HUNT* All-In Pain, Yurii S. Aulchenko[12,13], Knut Hagen[7,8], Egil A. Fors[9], Kristian Hveem[4,10], John-Anker Zwart[2,4,6], J.B.J. van Meurs[11], Maxim B. Freidin[1], Frances M.K. Williams[1*]

Affiliations:

1 Department of Twin Research and Genetic Epidemiology, School of Life Course Sciences, King's College London, London, United Kingdom
2 Department of Research, Innovation and Education, Division of Clinical Neuroscience, Oslo University Hospital, Oslo, Norway.
3 Department of Neurology, Oslo University Hospital, Oslo, Norway.
4 K. G. Jebsen Center for Genetic Epidemiology, Department of Public Health and Nursing, Faculty of Medicine and Health Sciences, Norwegian University of Science and Technology, Trondheim, Norway.
5 Research and Communication Unit for Musculoskeletal Health (FORMI), Department of Research, Innovation and Education, Division of Clinical Neuroscience, Oslo University Hospital, Oslo, Norway.
6 Institute of Clinical Medicine, Faculty of Medicine, University of Oslo, Oslo, Norway.
7 Department of Neuromedicine and Movement Science, Faculty of Medicine and Health Sciences, Norwegian University of Science and Technology (NTNU), Trondheim, Norway.
8 Clinical Research Unit Central Norway, St. Olavs University Hospital, Trondheim, Norway.
9 Department of Public Health and Nursing, Faculty of Medicine and Health Sciences, Norwegian University of Science and Technology, Trondheim, Norway.
10 HUNT Research Center, Department of Public Health and Nursing, Faculty of Medicine and Health Sciences, Norwegian University of Science and Technology, Trondheim, Norway.
11 Department of Internal Medicine, Erasmus Medical Center, Rotterdam, The Netherlands.
12 Laboratory of Theoretical and Applied Functional Genomics, Novosibirsk State University, Novosibirsk, Russia.
13 PolyOmica, ‘s-Hertogenbosch, 5237 PA, the Netherlands
14 Laboratory of Recombination and Segregation Analysis, Institute of Cytology and Genetics, 10 Lavrentiev Avenue, Novosibirsk, 630090, Russia

*Correspondence to frances.williams@kcl.ac.uk

Members of "**HUNT All-In Pain**"

Amy E Martinsen[1,2,3], Anne Heidi Skogholt[1], Ben Brumpton[1], Cristen Willer[4], Ingrid Heuch[2], Ingunn Mundal[5], Jonas Bille Nielsen[1,4,6], Kjersti Storheim[7,8], Kristian Bernhard Nilsen[9,10], Lars Fritsche[11], Laurent F. Thomas[1,12], Linda M Pedersen[2], Maiken E Gabrielsen[1], Marianne Bakke Johnsen[1,3,7], Marie Udnesseter Lie[3,7], Oddgeir Holmen[13], Synne Øien Stensland[7,14], Wei Zhou[15,16]

1 K. G. Jebsen Center for Genetic Epidemiology, Department of Public Health and Nursing, Faculty of Medicine and Health Sciences, Norwegian University of Science and Technology, Trondheim, Norway.

2 Department of Research, Innovation and Education, Division of Clinical Neuroscience, Oslo University Hospital, Oslo, Norway.

3 Institute of Clinical Medicine, Faculty of Medicine, University of Oslo, Oslo, Norway.

4 Department of Internal Medicine, Division of Cardiovascular Medicine, University of Michigan, Ann Arbor, 48109, MI, USA.

5 Department of Health Science, Molde University College, Molde, Norway.

6 Department of Epidemiology Research, Statens Serum Institut, Copenhagen, Denmark.

7 Research and Communication Unit for Musculoskeletal Health (FORMI), Department of Research, Innovation and Education, Division of Clinical Neuroscience, Oslo University Hospital, Oslo, Norway.

8 Faculty of Health Sciences, Department of physiotherapy, Oslo Metropolitan University, Oslo, Norway.

9 Department of Neuromedicine and Movement Science, Faculty of Medicine and Health Sciences, Norwegian University of Science and Technology (NTNU), Trondheim, Norway.

10 Department of Neurology, Oslo University Hospital, Oslo, Norway.

11 Center for Statistical Genetics, Department of Biostatistics, University of Michigan, Ann Arbor, 48109, MI, USA.

12 Department of Clinical and Molecular Medicine, Norwegian University of Science and Technology, Trondheim, Norway.

13 HUNT Research Center, Department of Public Health and Nursing, Faculty of Medicine and Health Sciences, Norwegian University of Science and Technology, Trondheim, Norway.

14 NKVTS, Norwegian Centre for Violence and Traumatic Stress Studies.

15 Department of Computational Medicine and Bioinformatics, University of Michigan, Ann Arbor, MI, USA.

16 Analytic and Translational Genetics Unit, Massachusetts General Hospital, Boston, Massachusetts, USA.

Online supplemental text

Supplemental methods
Supplemental results
Supplemental discussion

Online supplemental figures

Supplemental figure S1	Study flowchart of cases and controls
Supplemental figure S2	The QQ plot of GWAS summary statistics of chronic widespread musculoskeletal pain
Supplemental figure S3	Manhattan plot of sensitivity GWAS of chronic widespread musculoskeletal pain
Supplemental figure S4	Heatmap of genetic correlations for 23 complex traits with chronic widespread musculoskeletal pain
Supplemental figure S5	Hierarchical clustering of genetic correlations and heatmap of partial genetic correlations.
Supplemental figure S6	Functional consequences of candidate SNPs in genomic risk loci annotated by ANNOVAR
Supplemental figure S7	Colocalization of chronic widespread musculoskeletal pain associated locus (*RNF123*) with skeletal muscle and dorsal root ganglion eQTL
Supplemental figure S8	Prioritised genes at chronic widespread musculoskeletal pain associated *RNF123* locus

Online supplemental tables

Supplemental table S1	Genotyping and imputation methods across all cohorts.
Supplemental table S2	Independent SNPs significantly associated with chronic widespread musculoskeletal pain in UK Biobank
Supplemental table S3	Replication findings of three independent SNPs by cohort
Supplemental table S4	Sample-size based meta-analysis findings of replication SNPs
Supplemental table S5	Standard error based meta-analysis findings of replication SNPs
Supplemental table S6	Genetic correlations and partial genetic correlations between seven complex traits and chronic widespread musculoskeletal pain
Supplemental table S7	Differential gene set enrichment in 54 specific tissue types from GTEx
Supplemental table S8	Differential gene set enrichment in 30 general tissue types from GTEx
Supplemental table S9	Colocalization of *RNF123* locus with muscle skeletal eQTL signals
Supplemental table S10	Colocalization of *RNF123* locus with DRG eQTL signals at exon-level

References

Online supplemental text

Supplemental methods

Description of study cohorts

UKB. UKB is a population cohort comprising 502,682 individuals aged 40-73 years at recruitment, who are registered with a general practitioner within the UK National Health Service. Around 9.2 million individuals living within 25 miles of UKB assessment centre (n=22) located in England, Scotland, and Wales were invited to take part in the study between 2006 and 2010. Data collected was primarily self-reported. Participants were provided with touchscreen computer-based questionnaire and also attended a face-to-face interview administered by trained nurses. Each participant provided phenotypic and health-related information (e.g., pain, lifestyle and environmental) and biological samples (e.g., blood, urine and saliva). Following the Declaration of Helsinki, written informed consent was obtained from each participant[1]. UK Biobank's study protocol is available publicly (http://www.ukbiobank.ac.uk/wp-content/uploads/2011/11/UK-Biobank-Protocol.pdf?phpMyAdmin=trmKQlYdjjnQIgJ%2CfAzikMhEnx6) and research activities were reviewed and approved by the National Health Service National Research Ethics Service (ref.11/NW/0382). Majority of cohort participants (94%) self-reported to be white ancestry[2].

HUNT. The Nord-Trøndelag Health Study (HUNT) (https://www.ntnu.edu/hunt) is a population based, longitudinal study carried out in Nord-Trøndelag county in Norway. It comprises an ethnically homogenous, primarily Caucasian population. The study has been carried out in several waves (HUNT1-4), and in each survey, all inhabitants aged ≥ 20 years were invited to participate. A range of health-related data were obtained, both through questionnaires and clinical examinations. DNA from whole blood was collected in HUNT2 (1995-1997) and HUNT3 (2006-2008), with genotypes being available for 71,860 participants. Both surveys also included questions to define CWP[3]. A more detailed description of the HUNT Study is available elsewhere[4]. All study participants provided an informed, written consent to use their data and biological samples for research, and the study was approved by the Regional Committee of Medical and Health Research Ethics in Norway (REK #2015/573).

TwinsUK. TwinsUK cohort (www.twinsuk.ac.uk) comprises approximately 13,000 MZ and DZ twins aged between 18 to 93 living in the United Kingdom. TwinsUK registry commenced in 1992 and in later years additional twins were recruited to understand heritability, the genetic architecture of common diseases and the healthy ageing process. Participants of the TwinsUK cohort are predominantly females. Detailed phenotypic and omics data were collected from twins. All participants were recruited following the Declaration of Helsinki, and all research projects were approved by the Research Ethics Committee of the St. Thomas' Hospital. All participants of TwinsUK registry provided written consent. Information on CWP and other omics are available from the TwinsUK participants[5]. This study includes participants who responded to CWP questionnaire between 2002–2013.

RS. RS (www.epib.nl/research/ergo) is a population-based prospective cohort study in the district of Rotterdam, the Netherlands and comprised of three independent cohorts. The first cohort started in the 2nd half of 1989 with 7,983 persons aged ≥ 55 to 106 years living in Ommoord district in the city of Rotterdam called Rotterdam Study-1 (or RS-1). In the second cohort (Rotterdam Study-2 (or RS-2)), 3011 participants aged 55 in the year 1999 were added to the study. In the third cohort (Rotterdam Study-3 (RS-3)), 3932 participants aged between 45–54 years were added in the study. All three RS study participants were interviewed for 2 hours at home and extensively examined (e.g., imaging heart, blood vessels, eyes, skeleton and brain) for 5 hours in a research facility which was repeated in every 3 to 4 years in a research facility. Biospecimens were collected during the research facility visit. Informed consent was obtained from each participant, and the medical ethics committee of the Erasmus Medical Centre Rotterdam approved the study[6, 7].

ELSA. ELSA (https://www.elsa-project.ac.uk/) is a prospective open cohort comprised of a representative ageing population of England. This study was designed to capture the experience of the aged population in the 21st century. The study is ongoing but has collected a wide range of high-quality data in the last two decades, which includes health, economic, social, psychological, cognitive, biological and genetic data. At present, the ELSA study had completed eight waves (w-1 to w-8) of data collection between 2002-2017. In each wave, data was collected via

computer-assisted personal interview, a self-reported questionnaire, tests for cognitive function and walking speed. The nurse collected biological samples from participants. In the computer-assisted personal interview, along with other modules (e.g., household demographics, individual demographics, work and pensions), a health module was administered to all respondents which covered long-standing illness or disability, eyesight and hearing, specific diagnoses and symptoms, and pain etc. Ethical permission for all the ELSA waves was provided by the National Research Ethics Service (MREC/01/2/91)[8]. Use of ELSA data for this project was approved by METADAC data access committee (application reference: MDAC-2019-0928-03A-FREYDIN).

Phenotype definition – Discovery cohort

UKB. We defined Northern European ancestry if self-reported white-ancestry participants had similar genetic ancestry based on analysis of genetic principal components conducted centrally as recommended by UK Biobank (https://biobank.ctsu.ox.ac.uk/crystal/crystal/docs/ukb_genetic_data_description.txt).To define CWP, we initially used data fields (6159, 2956, 3799, 4067, 3404, 3571, 3741, 3414, 3773) from UKB phenotype file. The UKB participants were provided with a touchscreen questionnaire and asked, "In the last month have you experienced any of the following that interfered with your usual activities"? (data field=6159) Possible answers to choose from were 'none of the above'; 'prefer not to answer'; pain at seven different body sites (head, face, neck/shoulder, back, stomach/abdomen, hip, knee); or 'pain all over the body'. Unless reported "pain all over the body", participants could report more than one pain site. Those reported to have pain in the last month were further asked if the pain lasted for 3+ months (data field=2956, 3799, 4067, 3404, 3571, 3741, 3414, 3773). Participants with three months of "pain all over the body" were considered as cases of CWP (n=5,440). Also, those reported simultaneous pain in knee, shoulder, hip and back lasting for 3+ months were considered as cases of CWP (n=2,132). In addition, we used data field 20002 (Non-cancer illness) where participants either self-reported "fibromyalgia" or described the condition based on which the diagnosis was made by a healthcare professional. A total of 726 participants reported receiving a diagnosis of fibromyalgia which were included as cases. The exclusion of self-reported diagnosis of rheumatoid arthritis, polymyalgia rheumatica,

arthritis not otherwise specified, systemic lupus erythematosus, ankylosing spondylitis and myopathy was also based on data field 20002.

Phenotype definition – Replication cohorts

HUNT. The definition of CWP used in this study was published before [9]. In brief, participants were asked the screening question "Have you during the last year continuously for at least 3-months had pain and/or stiffness in muscles and joints?". Those who replied "yes" were requested to mark the location of nine pain sites (neck, shoulders, elbows, wrist/hands, upper back, low back, hips, knees, and/or ankles/feet). These nine anatomical pain sites were taken from the Nordic Questionnaire[10], and have been shown to be reliable in estimating low back and upper limb, and neck symptoms during the past year[11]. CWP cases were defined as those with pain located in the axial skeleton (neck, upper back, or lower back), above the waist (neck, shoulders, elbows, wrist/hands, or upper back), and below the waist (lower back, hips, knees, or ankles/feet). In HUNT3 cases were also required to have bilateral presence of the pain, but not in HUNT2, where no question on laterality was included. Controls were defined as participants who were free from any form of chronic musculoskeletal pain (< 3 months) in HUNT-2 and HUNT-3. Based on International Statistical Classification of Diseases (ICD)-10 codes participants with a diagnosis of rheumatoid arthritis, polymyalgia rheumatica, arthritis not otherwise specified (NOS), systemic lupus erythematosus, ankylosing spondylitis were excluded from the study. The final sample included in the replication analysis consisted of 10,556 CWP cases and 13,239 controls.

ELSA. Study participants were asked about their experience of pain using computer-assisted personal interview. Pain questions asked differed in their contents between the waves. In all waves, participants were asked "are you often troubled with pain?", following a "yes" response follow up questions were asked to identify the number of pain sites and severity and/or duration of pain. The methods of ascertaining pain sites differed between waves. In waves 1 and 2, participants were asked to report pain in 4 musculoskeletal sites (back, hip, knee and feet) on a scale of 0 (no pain) to 10 (severe excruciating pain). In contrast, in waves 3 to 8, participants were asked to report their experience of pain in the 7 sites (back pain, hip pain, knee pain, feet

pain, mouth pain, pain elsewhere and pain all over) and they could choose as many options as they liked. The severity of pain was asked in all waves, and duration of pain was requested explicitly in waves 4, 5 and 6. In the study, we defined CWP if participants reported pain all over or simultaneous pain in the back, hip and knee or back, hip, and feet or back, knee and feet which was lasting for more than three months or in their severity as moderate to severe (in the absence of pain duration). We defined controls if participants reported “No” to the question “are you often troubled with pain?”. Finally, we made a composite CWP binary variable by merging all cohorts where CWP cases identified in all waves served as cases. In contrast, controls were those found to be controls in any waves but never became cases in the earlier or later waves. A total of 1,679 cases and 5,304 controls with genotype data were included in the replication analysis.

TwinsUK. CWP information was collected on five occasions using questionnaires between 2002-2014. On three instances London Fibromyalgia Epidemiology Study Screening Questionnaire (LFESSQ)[12] was administered. In the other two instances broader information was collecting including site-specific questions or a mannequin was provided to report pain sites. Based on information collected, we defined CWP in the study as pain in the middle, left and right side of the body, above and below diaphragm lasting for three months or more[12]. A composite CWP variable was made by merging all five data collection time where cases were those ever reported to have CWP and controls were who did not fulfil the criteria of CWP in any of the waves. Participants with inflammatory diseases (n=67) and missing zygosity were excluded from the study. Participants who reported disabled low-back pain (n=455) were excluded from the controls. Finally, 1111 cases and 3556 controls with genotype data were included in the replication analysis.

RS. The RS study participants reported painful body sites (pain during at least half of the days during the last six weeks) using a pain homunculus. CWP was defined if participants reported pain sites in the left side of the body, in the right side of the body, above and below the waist, and in the axial skeleton. Same CWP definition was used in previous GWAS[7]. Controls were those reported no pain or any form of chronic musculoskeletal pain (≥ 3 months).

Genotyping and imputation methods

UKB participants were genotyped using Applied Biosystems UKB Axiom array and Applied Biosystems UKB Lung Exome Variant Evaluation Axiom array. HUNT participants were genotyped using Illumina/HumanCoreExome12 v1.0, Illumina/HumanCoreExome12 v1.1 and UM HUNT Biobank v1.0. ELSA participants were genotyped using the Illumina/HumanOmni2.5-4v1 and Illumina/HumanOmni2.5-8v1.3. TwinsUK participants were genotyped using Illumina/HumanHap300, Illumina/HumanHap610Q, Illumina/1M-Duo and 1.2MDuo 1M. RS-1 participants were genotyped using Illumina/HumanHap 550K V.3 and Illumina/HumanHap 550K V.3 DUO. RS-2 participants were genotyped using Illumina/HumanHap 550V.3DUO and Illumina/HumanHap610Q. RS-3 genotyping was performed using Illumina/HumanHap610Q. Imputation methods across cohorts are summarised in online supplemental table S1.

Selection of proxy SNPs in ELSA cohort

To identify proxy SNPs, we looked for ELSA genotyped SNPs around ±250kb of the discovery SNPs. We choose proxies for replication analysis using criteria that the SNP had minor allele frequency closer to the SNP identified in the discovery, showing highest R^2 (> 0.80) with the discovered SNP and had lowest genetic association p-value in the discovery cohort. The *rs9870858* and *rs17329848* have been considered as best proxy SNPs for *rs1491985* and *rs10490825*, respectively.

Statistical analysis and in-silico follow-up

Discovery association analysis

We applied the following filters to discovery analysis: minor allele frequency (MAF) ≥ 0.01, imputation quality scores (INFO) ≥ 0.70, SNPs and individuals missingness rates not exceeding 0.02. Plink V.2[13] has been used to determine SNPs passing Hardy-Weinberg equilibrium (HWE) threshold p > 1E-06. P-value threshold 5E-08 was used to declare GWAS significance. Association analysis was performed using BOLT-LMM (v2.3.2), which accounts for population structure and cryptic relatedness [14].

Identification of independent SNPs

To identify independent SNPs located in GWAS significant loci (p<5E-08) we used multi-SNP-based conditional & joint association (COJO)[15] analysis implemented in software package GCTA[16]. A stepwise model selection procedure was used to identify independently associated SNP by conditioning on other significant SNPs at the locus. SNPs with minor allele frequency ≤ 0.01 was excluded. Randomly selected 50,000 European ancestry participants from the UK Biobank were used as LD reference sample for the COJO analysis. In addition to COJO, we used Functional Mapping and Annotation of genetic associations (FUMA) v1.3.4[17] to identify independent SNPs at p<5E-08 by examining the relationship between independent SNPs at r2 < 0.1. The 1000 genome phase-3 European ancestry data was used as a reference panel to define LD blocks (<250 kb apart, MAF ≥ 0.01). Findings of GCTA-COJO and FUMA were identical.

Replication and Meta-analysis

HUNT Association testing. We performed association testing between independent SNPs and CWP using the Scalable and Accurate Implementation of Generalized mixed model (SAIGE)[18], which uses a generalized mixed model to account for sample relatedness and cryptic population structure. We performed a mixed-effects linear regression model, including age, sex, genotype batch, and the first four genetic principal components as covariates.

TwinsUK association testing. We performed a linear mixed-effects model using Genome-wide Efficient Mixed Model Association (GEMMA) v0.98.1[19] to estimate the effect of each independent SNP. Regression models were adjusted for age, sex, and the genetic relatedness matrix.

ELSA association testing. We performed a mixed-effects linear model using Genome-wide Complex Trait Analysis (GCTA) v1.91.7 beta1[16] to estimate the effect of each independent SNPs. Regression models were adjusted for age, sex, and the genetic relationship matrix.

RS association testing. For all three RS cohorts, we performed a linear regression model using PLINK v1.9.[13] to estimate the effect of each independent SNPs. Assuming homogenous study population, RS cohorts were adjusted for age and sex only.

Meta-analysis of replication cohorts. Association findings of each SNP across all replication cohorts were meta-analysed using fixed effects model with sample size and inverse-variance weighting implemented in METAL[20]. Between-study heterogeneity was assessed using I^2 statistics. Multiple testing correction was applied to declare significance following meta-analysis (0.05/3= 0.017). We performed both sample size and standard error based meta-analysis. Power calculation showed that replication meta-analysis power for three independent SNPs ranges between 46.3 to 49.7%.

Genomic inflation, heritability, genetic correlation and partial genetic correlations

LD score regression (LDSR)[21] was used to assess inflation (λ_{GC}) in test statistics and to distinguish confounding from polygenicity. We also used BOLT-REML to estimate SNP-heritability of CWP[22]. Observed scale SNP-heritability was converted on the liability scale assuming a *CWP* prevalence of 2.8% in the sample and population. We measured the genetic correlation (GC) between CWP and 209 complex traits from LD-hub [23] using LDSR tools[21]. LD-hub database includes 597 UKB traits from Benjamin Neale's group generated without rigorous quality control for many phenotypes. Therefore, we choose not to use those summary statistics for the estimation of genetic correlations. Precomputed LD scores using 1000 Genomes European data restricted to HapMap3 SNPs (n=1,217,311) were used to calculate both SNP heritability and genetic correlations. Precomputed LD scores and the list of HapMap3 SNPs were obtained from https://data.broadinstitute.org/alkesgroup/LDSCORE/.
Bonferroni-corrected p-value < 0.01/209 = 4.78E-05 was used to declare significance for GC analysis. Based on hierarchical clustering, we identified 7 clusters of genetically correlated traits, of which seven representative traits were chosen for partial GC analysis. Partial GC quantifies the proportion of GC, which is not influenced by other traits. Visualization of GC, hierarchical clustering and partial GC implemented in R using package "corrplot" with basic "hclust" function. Bonferroni-corrected p-value < 0.01/7 = 0.001 was used to declare significance for partial GC analysis.

Functional annotation of CWP associated SNPs

To identify the functional consequences of GWAS independent SNPs at p <5E-08, we used ANNOVAR[24] implemented in FUMA[17]. Independent SNPs identified at r^2<0.6 within a

250kb window and their LD proxies with MAF ≥ 1% were selected using 1000 Genomes Project Phase 3 as a reference panel. All independent SNPs and proxy SNPs were taken forward for annotation in ANNOVAR with Ensembl genes build v92. Additionally, CADD score (a score >12.37 considered to be pathogenic), RegulomeDB (RDB) scores (which ranges from 1 to 7 where the lower score indicates a higher likelihood of having a regulatory function), and 15-core chromatin states (chromatin state <8 indicates an open chromatin region with higher accessibility as the score decreases) were annotated. All these features were embedded in the FUMA web tool.

Gene mapping

We used four different strategies (genome-wide gene-based association analysis, positional, eQTL, and chromatin interaction mapping) for gene mapping. MAGMA (Multi-marker Analysis of GenoMic Annotation) v1.07[25] was used for gene-based genome-wide association analysis (GWGAS), which was implemented in a web tool FUMA[17] v1.3.6. In GWGAS analysis, SNPs from the CWP GWAS summary statistics were mapped to 19261 protein-coding genes using gene definition of NCBI Build 37/UCSC hg19. All SNPs locating within ±50Kb of the gene body were used to calculate a gene test-statistics (p-value) using default SNP-wide mean model. The major histocompatibility complex (MHC) region was excluded from the analysis. For the calculation of LD 1000 genome phase-3 European ancestry data was used as a reference. Results were presented with Bonferroni correction to control for multiple testing ($P < 0.05/19{,}261= 2.6E-6$).

For the positional gene mapping, ANNOVAR annotated SNPs were mapped to protein-coding genes within 10kb window from the human reference assembly (GRCh37/hg19) using FUMA. For the eQTL mapping, all independent SNPs and proxy SNPs identified by FUMA were mapped to all eQTL data repositories available in the FUMA with default settings. All SNPs were mapped to genes where the allelic variation of SNP affects the expression level of those genes up to 1 Mb. An FDR (false discovery rate) threshold < 0.05 was used to define significant eQTL association. In the chromatin interaction mapping, all candidate SNPs were mapped to genes' promoter regions (defined with a window of 250bp upstream and 500bp downstream of TSS) based on significant chromatin interaction. This mapping strategy does not

require distance boundary; therefore, genes located in long-distance can be mapped. When an independent SNP is located in a region interacting with a region containing several genes, then all of those genes were mapped with that SNP. We used Hi-c data of 21 tissues and cell types from GSE87112 available in FUMA by default for chromatin interaction mapping. To prioritise candidate genes, we performed the filtering of candidate SNPs overlapping with enhancers and promoters predicted from 111 tissue/cell types from the Roadmap Epigenomics Project. This strategy reduces gene number and increases the likelihood that the remaining genes are biologically relevant. An FDR <1E-06 were used to detect significant interaction.

Tissue specificity and gene-set enrichment analyses

Tissue and gene set enrichment analyses were conducted with GENE2FUNC, an integrated process of FUMA[17] web tool. A total of 89 mapped genes identified by GWGAS, positional, eQTL or chromatin interaction mapping were used as input. Tissue specificity for 54 specific tissues and 30 general tissues obtained from the GenotypeTissue Expression (GTEx) v8 database were tested using previously defined differentially expressed gene (DEG) sets. All mapped genes were tested against each DEG sets with the hypergeometric test. Additionally, an overrepresentation of mapped genes in any of the well-defined hallmark gene sets available in the molecular signature database (MsigDB) were tested. Tissue specificity and gene-set enrichment were conducted using FDR adjusted p-value threshold <0.05 and minimum overlapping genes with gene-sets ≥2. All genes available by default were used as background gene-set for the enrichment analysis. All of these analyses were performed, excluding the MHC genomic region.

Colocalization analyses using skeletal muscle and dorsal root ganglia eQTLs

We aimed to explore the cis-regulation of CWP associated variants in both skeletal muscle (n=706) and human DRG (n=214) using publicly available eQTL data (skeletal muscle: https://gtexportal.org/home/; DRG: http://diatchenko.lab.mcgill.ca/DRG-eQTLs/). SNPs regulating the expression level of a gene known as eQTLs. Cis-acting eQTLs were located within ≤1Mb of the transcription start site of the target gene [26]. Details of skeletal muscle and DRG eQTLs are available here[27, 28]. We extracted the summary statistics of SNPs associated with CWP at 1E-05 and located within a 200-kb window around GWAS independent SNPs. Extracted SNPs overlapping with skeletal muscle and DRG eQTLs were used for colocalization. Before colocalization analysis with GTEx skeletal muscle eQTLs, CWP-GWAS

associated RSIDs were aligned to the human reference genome build GRCh38 using LiftOver tool (https://genome.ucsc.edu/cgi-bin/hgLiftOver). We applied Bayesian colocalization method (coloc)[29] with CWP prevalence and "cc" trait type as parameters to integrate CWP-GWAS with skeletal muscle and DRG cis-eQTLs data assuming a single causal variant underlying the locus. Colocalization of skeletal muscle eQTLs was assessed at gene-level. For DRG, both gene- and exon-level cis-eQTLs were assessed for colocalization. A total of five hypotheses were tested to evaluate colocalization, H0: there is no causal variant for both traits); H1 or H2: causal variant associated with either trait-1 or trait-2, H3: two independent causal variants for trait-1 and trait-2; and H4: one single causal variant associated with both traits. Coloc generates higher posterior probability (PP) to test each hypothesis. A higher posterior probability for H3 (PP3) supports the presence of two independent variants for both traits. A higher posterior probability for H4 (PP4) supports the presence of single independent variants affecting both traits. We reported eQTL SNP at the locus having lowest p-value as evidence of colocalization.

Functional annotation of *RNF123* locus

FUMA mapped genes at *RNF123* locus were submitted to the open targets platform (https://www.targetvalidation.org/) to prioritise candidate gene in this locus. Two genes (*RP11-3B7.1* and *CTD-2330K9.3*) located in the locus were not recognised by the platform. Therefore, 53 genes were assessed for the relevance of these genes with musculoskeletal or connective tissue disease. We considered the gene-set with the lowest p-value for gene prioritisation. A gene-set containing 45 genes were associated with musculoskeletal system diseases with a p-value=2E-14. Disease-associated genes obtained a score ranging between 0 to 1, where 0 indicate no evidence of association and the higher the association score indicate stronger evidence of association (highest possible score is 1). Each of the genes received scoring in seven data types (genetic association, somatic mutations, drugs, pathway and systems biology, RNA expression, text mining and animal models). Scores from all these data types were aggregated to calculate an overall association score (details of scoring available here: https://docs.targetvalidation.org/getting-started/scoring). Genes with the highest overall association score equal to 1 were prioritised as putative causal genes in the locus.

Supplemental results

Functional consequences of SNPs

Independent SNPs and their proxies were annotated for functional consequences for using ANNOVAR. In total, 225 candidate SNPs were used for annotation. The results of ANNOVAR annotation presented in online supplemental table S6 and online supplemental figure S6. Majority of the annotated SNPs were intronic (83.6%). None of the annotated SNPs were non-synonymous. We found 5 synonymous variants located in genes *ATP2C1 (rs16835513), BSN (rs4855885), MST1 (rs3020779), TRAIP (rs35129566)* and *ARVCF (rs2073747)*. A total of 4% (n=9) of annotated SNPs having CADD score >12.37 indicating deleterious nature of these SNPs, of which three SNPs *(rs62280752, rs28362548* and *rs62282192)* were located at gene *ATP2C1*. An RDB score <2 was observed for 9.3% (n=21) of the SNPs indicate that these variants are likely to regulate gene expression. Finally, 96% of the annotated SNPs had minimum chromatin state <8 indicate additional evidence for the regulatory potential of these SNPs.

Supplemental discussion

Generalizability to homogenous population

UKB collected data from people who were 40-69 years old and achieved a response rate of 5.47% [30]. It is known that UKB manifests healthy participants bias, which is likely the reason for the prevalence of CWP (2.8%) in UKB being considerably lower than that of the general UK population (14.2%). It is unlikely that lower prevalence is due to the genotype-dependent survival bias. If study non-participation is dependent on genotype, then the study will be biased towards the null. However, a simulation study had shown that genotype-dependent survival bias had little influence on the effect size when the study participants were <75 years old [31]. As UKB Biobank recruited participants <75 years it is unlikely that genotype-dependent survival bias has impacted our findings. Also, generalizing GWAS findings depends on reproducibility[32] and we have used independent cohorts, many from outside the UK, for the replication of GWAS findings. Therefore, we believe generalizability of our GWAS findings possible to northern Europeans.

Online supplemental figures

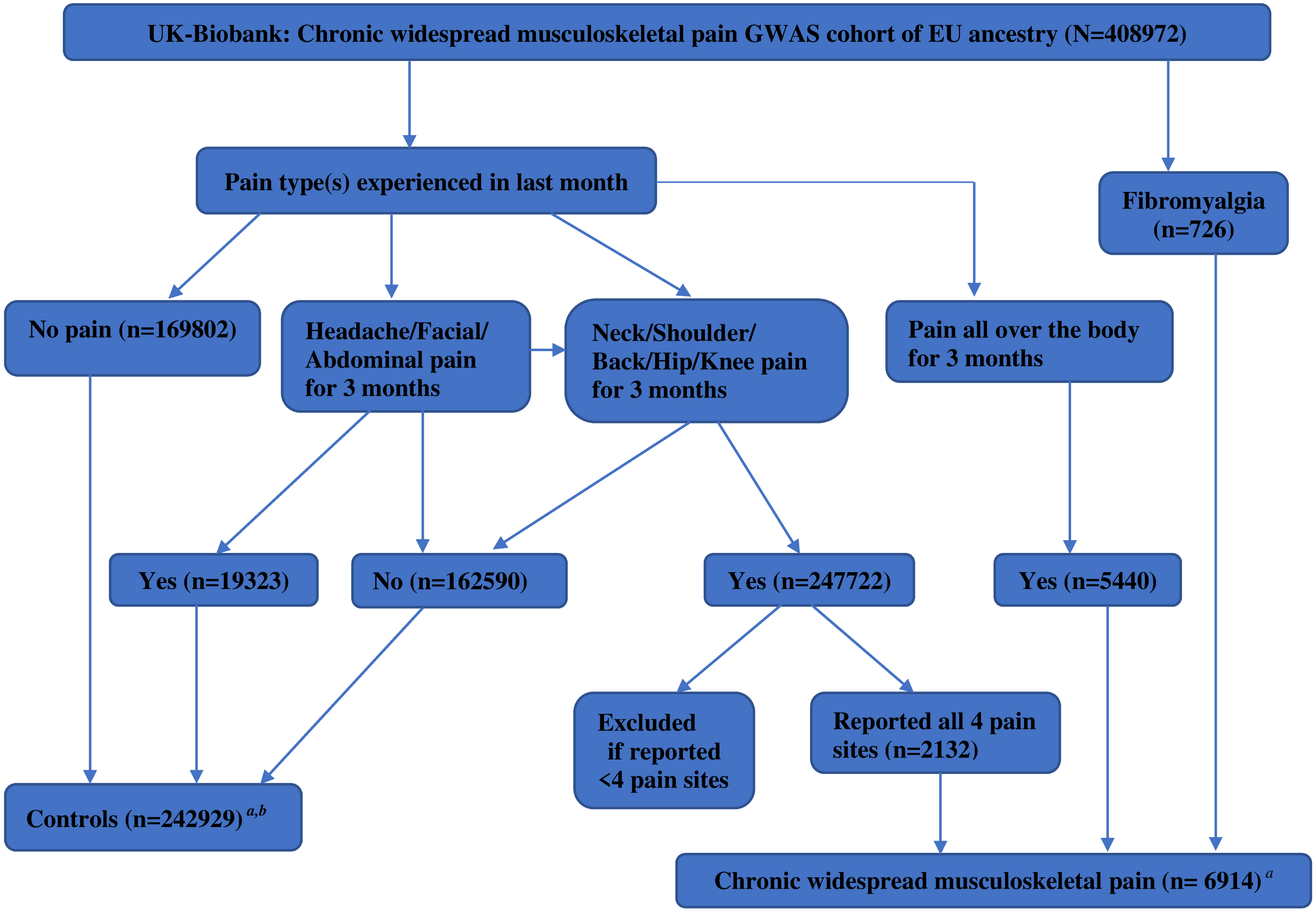

Supplemental figure S1. Study flowchart of cases and controls. UK-Biobank data fields 6159, 2956, 3799, 4067, 3404, 3571, 3741, 3414, 3773 and 20002 were used to define cases of chronic widespread musculoskeletal pain and controls. GWAS, genome-wide association study.

[a]Excluded participants with self-reported rheumatoid arthritis (n=4766), polymyalgia rheumatica (n=947), arthritis not otherwise specified (n=3828), systemic lupus erythematosus (n=455), ankylosing spondylitis (n=1187) and myopathy (n = 154).

[b]Total number of controls (242929) is less than the combined number of 169802+19323+162590 due to sample overlap between non-musculoskeletal (headache/facial/abdominal pain) and musculoskeletal pain (neck/shoulder/back/hip/knee pain) responders and the exclusion of diagnostic confounders from the samples as specified in footnote [a].

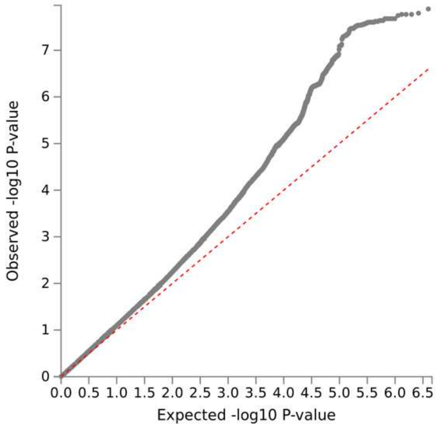

Supplemental figure S2. The QQ plot of GWAS summary statistics of chronic widespread musculoskeletal pain derived from UK biobank European ancestry data. The x-axis displays the expected – log10 transformed p-values and the y-axis displays the observed –log10 transformed p-values.

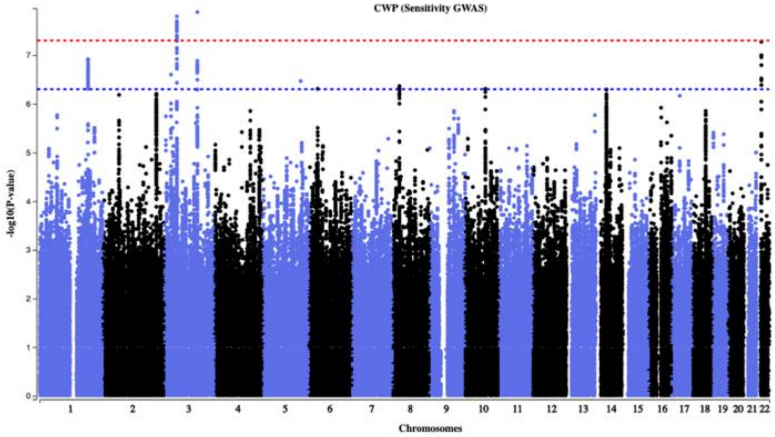

Supplemental figure S3. Manhattan plot of sensitivity GWAS of chronic widespread musculoskeletal pain, which excluded participants who reported chronic non-musculoskeletal pain.

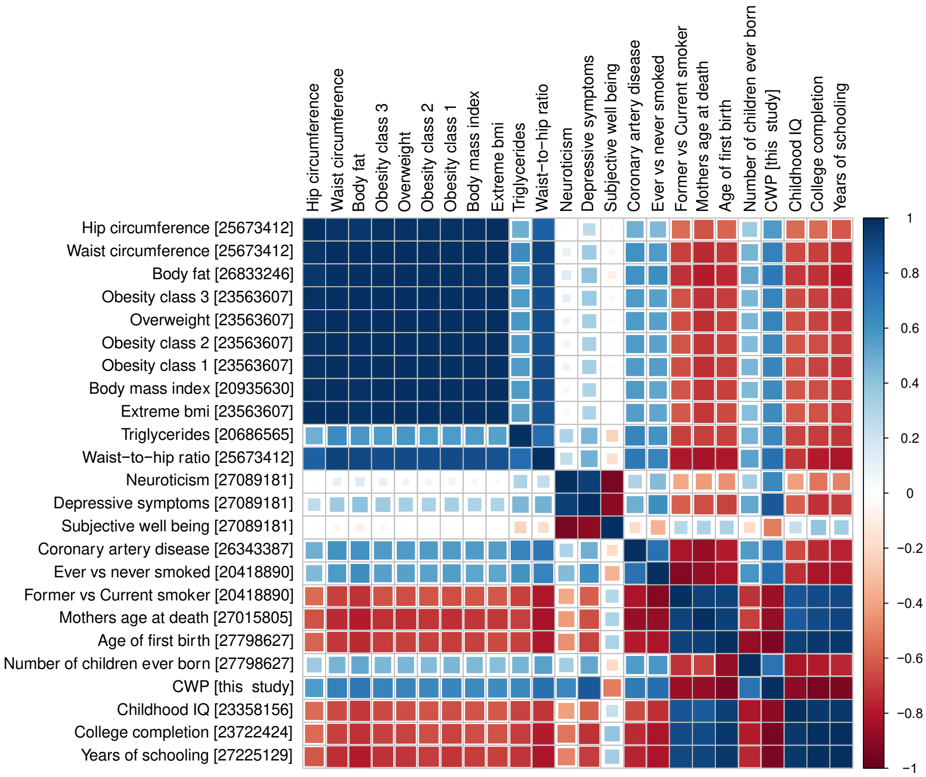

Supplemental figure S4. Heatmap of genetic correlations for 23 complex traits with chronic widespread musculoskeletal pain (CWP) (absolute $r_g \geq 0.20$; $p < 4.78E-05$). Each coloured cell indicates magnitudes of genetic correlations. The corresponding colour scale is presented on the right side of the heatmap where dark blue represents the highest genetic correlation, and darker red represents highest negative correlation. CWP, chronic widespread musculoskeletal pain. On the y-axis, PMID references for each complex trait are placed in the square brackets. On the x-axis, all complex traits are presented maintaining the order of the y-axis.

A. B.

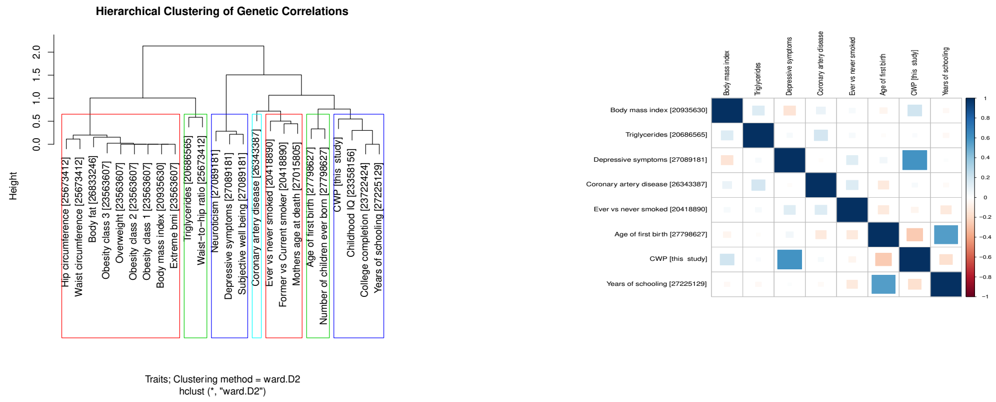

Supplemental figure S5. (A) Hierarchical clustering of genetic correlations for all pairs of traits. PMID references are placed in square brackets. Each cluster indicated with a coloured box. A total of 7 clusters were identified and (B) Heatmap of partial genetic correlations for 7 complex traits with chronic widespread musculoskeletal pain (CWP). Each coloured cell indicates magnitudes of genetic correlations. The corresponding colour scale is presented on the right side of the heatmap where dark blue represents the highest genetic correlation, and darker red represents highest negative correlation. CWP, chronic widespread musculoskeletal pain. On the y-axis, PMID references for each complex trait are placed in the square brackets. On the x-axis, all complex traits are presented maintaining the order of the y-axis.

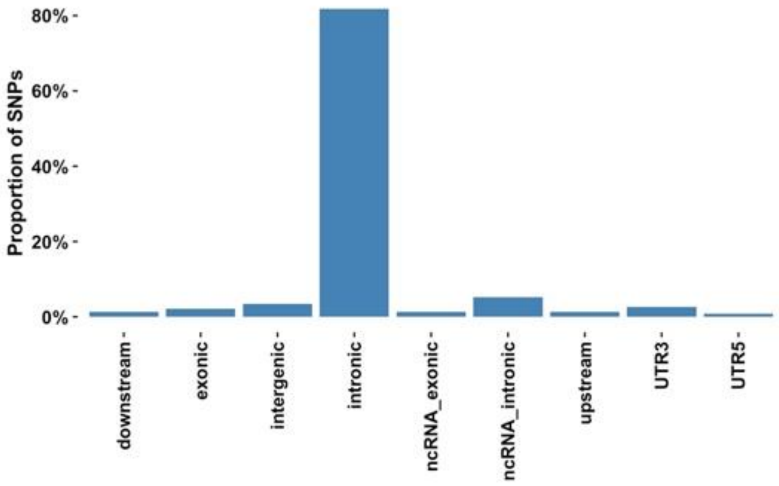

Supplemental figure S6. Functional consequences of candidate SNPs in genomic risk loci annotated by ANNOVAR

A. **B.**

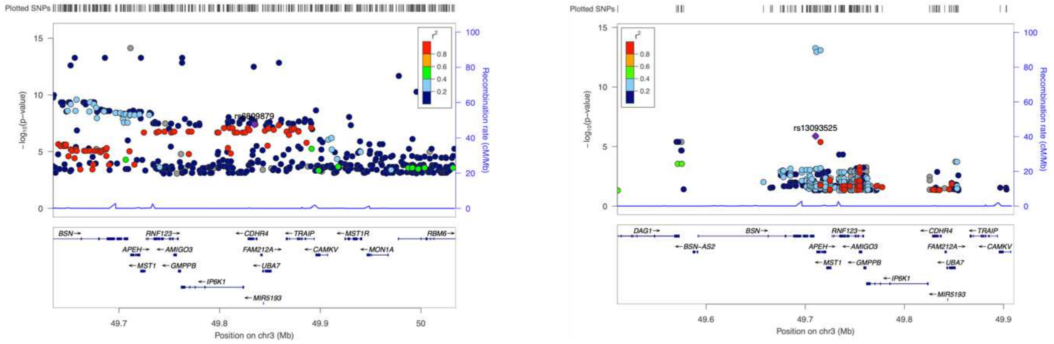

Supplemental figure S7. Colocalization of chronic widespread musculoskeletal pain associated locus (*RNF123*) with (A) Skeletal Muscle eQTL (gene-level) and (B) Dorsal root ganglion eQTL (exon-level). Independent SNPs are coloured in purple. Other coloured circles indicated pairwise LD. Strength of LD (r^2) presented in the upper right corner of each plot.

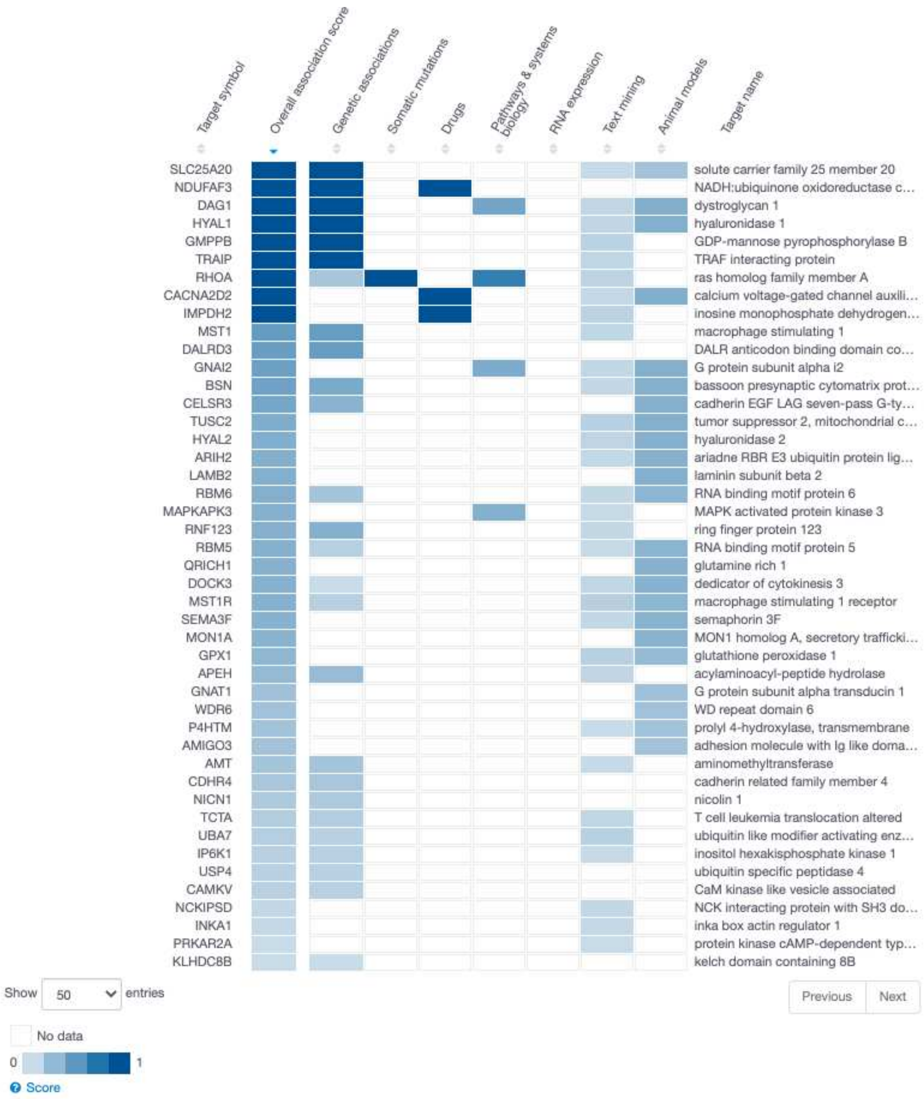

Supplemental figure S8. Prioritised genes at chronic widespread musculoskeletal pain associated *RNF123* locus. Genes with the highest overall association score=1 were prioritised (highlighted in dark blue). Scoring details are available here, https://docs.targetvalidation.org/getting-started/scoring. This plot was created on the open target platform (https://www.targetvalidation.org/).

Online supplemental tables

Supplemental table S1: Genotyping and imputation methods across all cohorts.

	Genotyping platform	Imputation procedure	Reference population
**Discovery Cohort**			
UK Biobank	Applied Biosystems UKB Axiom array, and Applied Biosystems UKB Lung Exome Variant Evaluation Axiom array	IMPUTE 4<br><br>MHC: HLA was imputed separately; using HLA*IMP:02 algorithm with a multi-population reference panel.	Haplotype Reference Consortium (HRC), UK10K &1000 genome panel
**Replication cohorts**			
Twins UK	Illumina/HumanHap300, Illumina/HumanHap610Q, Illumina/1M-Duo, and Illumina/1.2MDuo 1M	MACH	1000G Phase3 v5
RS-1	Illumina/HumanHap 550K V.3 and Illumina/HumanHap 550K V.3 DUO	MACH	HapMap release 22 CEU
RS-2	Illumina/HumanHap 550V.3DUO, and Illumina/HumanHap610Q	MACH	HapMap release 22 CEU
RS-3	Illumina/HumanHap610Q	MACH	HapMap release 22 CEU
HUNT	Illumina/HumanCoreExome12 v1.0, Illumina/HumanCoreExome12 v1.1, and UM HUNT Biobank v1.0	Minimac3 (v2.0.1)	Haplotype Reference Consortium, and HUNT-specific WGS
ELSA	Illumina HumanOmni2.5 Bead Chips (HumanOmni2.5-4v1, and HumanOmni2.5-8v1.3)	MACH	Haplotype Reference Consortium

Supplemental table S2. Independent SNPs significantly associated with chronic widespread musculoskeletal pain in UK Biobank.

SNP	CHR:BP	A1	A2	A1FREQ	INFO	BETA	SE	p-value	OR, 95% CI	Nearest gene
rs1491985	3:49739507	G	C	0.18	1	0.0034	0.0006	1.60E-08	1.13, 1.09-1.17	*RNF123*
rs10490825	3:130696383	G	A	0.87	1	-0.0039	0.0007	1.30E-08	0.87, 0.81-0.93	*ATP2C1*
rs165599	22:19956781	G	A	0.30	1	-0.0028	0.0005	2.50E-08	0.90, 0.86-0.94	*COMT/ARVCF*

Describe the model and adjustment The independent SNPs at locus reported with RSID, genomic coordinates (CHR:BP; GRCh37.p13/ Hg19/); A1, effect allele; A2, other allele; A1FREQ, effect allele frequency; INFO, estimated imputation score, Beta, linear regression coefficient; SE, standard error; OR, odds ratio; CI, confidence interval, Beta and standard errors of each SNP were divided by (μ * (1 - μ)) to obtain log ORs, where μ represents case fraction.

Supplemental table S3. Replication findings of three independent SNPs by cohort.

SNP	CHR	BP	A1	A2	A1FREQ	BETA	SE	p-value	N (cases)	Power	INFO
**TWINS UK**											
rs1491985	3	49739507	C	G	0.83	-0.0348	0.0122	0.0044	4667 (1111)	5.46%	1
rs10490825	3	130696383	A	G	0.13	0.0091	0.0141	0.5176	4667 (1111)	5.17%	0.99
rs165599	22	19956781	A	G	0.69	0.0066	0.0105	0.5290	4667 (1111)	5.30%	0.99
**HUNT**											
rs1491985	3	49739507	C	G	0.8279	-0.0107	0.0062	0.0844	23795 (10556)	26.75%	0.99
rs10490825	3	130696383	A	G	0.1435	0.0140	0.0067	0.0367	23795 (10556)	24.72%	0.99
rs165599	22	19956781	A	G	0.7196	0.0026	0.0052	0.6162	23795 (10556)	25.68%	Genotyped
**ELSA**											
rs9870858*	3	49769071	C	T	0.1867	0.0199	0.0092	0.0312	6983 (1679)	7.64%	Genotyped
rs17329848*	3	130590962	C	T	0.1213	-0.0004	0.0110	0.9739	6983 (1679)	7.17%	Genotyped
rs165599	22	19956781	G	A	0.3049	-0.0015	0.0077	0.8433	6983 (1679)	7.39%	Genotyped
**RS 1**											
rs1491985	3	49739507	C	G	0.8171	-0.0320	0.0899	0.7216	3136 (532)	4.12%	0.98
rs10490825	3	130696383	A	G	0.1296	0.1409	0.0990	0.1549	3136 (532)	3.94%	1
rs165599	22	19956781	A	G	0.7224	-0.0042	0.0769	0.9560	3136 (532)	4.03%	1
**RS 2**											
rs1491985	3	49739507	C	G	0.8077	-0.01654	0.1555	0.9153	1565 (144)	2.84%	1
rs10490825	3	130696383	A	G	0.1383	0.005822	0.1783	0.974	1565 (144)	2.76%	1
rs165599	22	19956781	A	G	0.7137	-0.08953	0.136	0.5103	1565 (144)	2.80%	1
**RS 3**											
rs1491985	3	49739507	C	G	0.8047	-0.2453	0.1381	0.0758	2934 (155)	3.95%	1
rs10490825	3	130696383	A	G	0.1377	0.0877	0.1685	0.6027	2934 (155)	3.78%	0.99
rs165599	22	19956781	A	G	0.7217	-0.0905	0.1303	0.4874	2934 (155)	3.86%	0.99

Replication SNPs at locus reported with RSID, genomic coordinates (CHR:BP; GRCh37.p13/ Hg19/); A1, effect allele; A2, Alternative allele; A1FREQ, effect allele frequency; Beta, linear regression coefficient; SE, standard error; N, sample size used of each SNP analysis; INFO, Imputation score; HUNT: The Nord-Trøndelag Health Study; ELSA: The English Longitudinal Study of Ageing; RS-1, 2 and 3: The Rotterdam Study 1,2,3.
*rs9870858 (instead of rs1491985) and rs17329848 (instead of rs10490825) were used as proxy in the ELSA cohort.

Supplemental table S4. Sample-size based meta-analysis findings of independent SNPs.

SNP	A1	A2	A1Freq	FreqSE	Weight	Z	P-value	Direction	$HetI^2$	$HetChi^2$	HetPVal	Power
rs1491985*	C	G	0.82	0.008	43080	-3.667	0.0002	------	10.20	5.57	0.35	49.70
rs10490825*	A	G	0.14	0.0083	43080	2.278	0.0227	++-+++	0	1.89	0.86	46.32
rs165599	A	G	0.71	0.0124	43080	0.338	0.7356	+++---	0	1.49	0.91	47.93

A1Freq: Frequency of A1 allele; FreqSE: Standard error for the frequency of A1 allele; Z: Z statistics; $HetI^2$: Heterogeneity I^2 parameter; $HetChi^2$: Heterogeneity test statistic; HetPVal: P-value for heterogeneity statistic.

* rs9870858 (instead of rs1491985) and rs17329848 (instead of rs10490825) were used as proxy in the ELSA cohort.

Supplemental table S5. Standard error based meta-analysis findings of independent SNPs.

SNP	A1	A2	A1Freq	FreqSE	Effect	StdErr	P-value	Direction	$HetI^2$	$HetChi^2$	HetPVal
rs1491985*	C	G	0.82	0.0064	-0.0171	0.0047	0.0003	------	17	6.03	0.30
rs10490825*	A	G	0.14	0.0093	0.0104	0.0053	0.049	++-+++	0	3.21	0.67
rs165599	A	G	0.71	0.0134	0.0027	0.004	0.50	+++---	0	1.14	0.95

A1Freq: Frequency of A1 allele; FreqSE: Standard error for the frequency of A1 allele; StdErr: Standard Error, $HetI^2$: Heterogeneity I^2 parameter; $HetChi^2$: Heterogeneity test statistic; HetPVal: P-value for heterogeneity statistic.

* rs9870858 (instead of rs1491985) and rs17329848 (instead of rs10490825) were used as proxy in the ELSA cohort.

Supplemental table S6. Genetic and partial genetic correlations for chronic widespread musculoskeletal pain with body mass index, triglycerides, depressive symptoms, coronary artery disease, ever vs never smoked, age of first birth and years of schooling.

	Genetic correlations			Partial genetic correlations	
	rg	SE	P	Partial rg	P
Body mass index [20935630]	0.31	0.0358	8.81E-18	0.20	2.36E-08
Triglycerides [20686565]	0.20	0.0462	1.10E-05	0.02	0.6571
Depressive symptoms [27089181]	0.65	0.0516	2.06E-36	0.59	9.22E-31
Coronary artery disease [26343387]	0.25	0.0397	5.38E-10	0.03	0.4293
Ever vs never smoked [20418890]	0.27	0.0588	5.83E-06	-0.05	0.3802
Age of first birth [27798627]	-0.58	0.0412	2.03E-44	-0.26	3.24E-10
Years of schooling [27225129]	-0.54	0.0331	4.23E-60	-0.17	3.37E-07

rg, genetic correlation estimate; SE, standard error; P, p-value; partial rg, partial genetic correlation estimate.

Supplemental table S7. Differential gene set enrichment in 54 specific tissue types from GTEx.

Category	GeneSet	N_genes	N_overlap	p	adjP	genes
DEG.twoside	Muscle_Skeletal	7979	51	3.27E-10	1.76E-08	ENSG00000115365:ENSG00000100075:ENSG00000070371:ENSG00000184058:ENSG00000215012:ENSG00000093010:ENSG00000099889:ENSG00000128191:ENSG00000099899:ENSG00000099901:ENSG00000099904:ENSG00000213672:ENSG00000114302:ENSG00000178537:ENSG00000177479:ENSG00000178467:ENSG00000178149:ENSG00000198218:ENSG00000177352:ENSG00000188315:ENSG00000114316:ENSG00000145022:ENSG00000145020:ENSG00000145029:ENSG00000173531:ENSG00000164068:ENSG00000173540:ENSG00000176095:ENSG00000185614:ENSG00000182179:ENSG00000183763:ENSG00000004534:ENSG00000001617:ENSG00000114353:ENSG00000243477:ENSG00000114378:ENSG00000068001:ENSG00000007402:ENSG00000114735:ENSG00000114738:ENSG00000088538:ENSG00000196455:ENSG00000017260:ENSG00000034533:ENSG00000198585:ENSG00000138246:ENSG00000240303:ENSG00000113971:ENSG00000125388:ENSG00000004864:ENSG00000092964
DEG.down	Brain_Hippocampus	7493	48	1.78E-09	9.63E-08	ENSG00000021826:ENSG00000070371:ENSG00000070010:ENSG00000215012:ENSG00000099889:ENSG00000183597:ENSG00000128191:ENSG00000099899:ENSG00000099901:ENSG00000099904:ENSG00000234409:ENSG00000099917:ENSG00000114302:ENSG00000178537:ENSG00000177479:ENSG00000178252:ENSG00000178149:ENSG00000198218:ENSG00000172037:ENSG00000177352:ENSG00000185909:ENSG00000188315:ENSG00000114316:ENSG00000145020:ENSG00000173402:ENSG00000164062:ENSG00000173531:ENSG00000164068:ENSG00000173540:ENSG00000185614:ENSG00000182179:ENSG00000183763:ENSG00000001617:ENSG00000214706:ENSG00000243477:ENSG00000114378:ENSG00000068001:ENSG00000007402:ENSG00000114735:ENSG00000114738:ENSG00000196455:ENSG00000034533:ENSG00000114686:ENSG00000138246:ENSG00000240303:ENSG00000113971:ENSG00000124664:ENSG00000004864
DEG.down	Whole_Blood	6908	45	5.47E-09	2.95E-07	ENSG00000115365:ENSG00000100075:ENSG00000070371:ENSG00000100084:ENSG00000215012:ENSG00000093010:ENSG00000099889:ENSG00000128191:ENSG00000099899:ENSG00000099901:ENSG00000099904:ENSG00000213672:ENSG00000114302:ENSG00000177479:ENSG00000178467:ENSG00000178252:ENSG00000178149:ENSG00000198218:ENSG00000172037:ENSG00000177352:ENSG00000185909:ENSG00000145020:ENSG00000145029:ENSG00000173402:ENSG00000164062:ENSG00000173531:ENSG00000173540:ENSG00000185614:ENSG00000164077:ENSG00000004534:ENSG00000214706:ENSG00000243477:ENSG00000068001:ENSG00000114383:ENSG00000007402:ENSG00000114735:ENSG00000196455:ENSG00000017260:ENSG00000034533:ENSG00000114686:ENSG00000138246:ENSG00000240303:ENSG00000113971:ENSG00000004864:ENSG00000092964
DEG.down	Muscle_Skeletal	6836	44	1.39E-08	7.50E-07	ENSG00000115365:ENSG00000100075:ENSG00000215012:ENSG00000093010:ENSG00000099889:ENSG00000128191:ENSG00000099899:ENSG00000099901:ENSG00000099904:ENSG00000213672:ENSG00000178467:ENSG00000178149:ENSG00000198218:ENSG00000177352:ENSG00000188315:ENSG00000114316:ENSG00000145022:ENSG00000145020:ENSG00000145029:ENSG00000173531:ENSG00000173540:ENSG00000176095:ENSG00000185614:ENSG00000182179:ENSG00000183763:ENSG00000004534:ENSG00000001617:ENSG00000114353:ENSG00000243477:ENSG00000114378:ENSG00000068001:ENSG00000007402:ENSG00000114735:ENSG00000088538:ENSG00000196455:ENSG00000017260:ENSG00000034533:ENSG00000198585:ENSG00000138246:ENSG00000240303:ENSG00000113971:ENSG00000125388:ENSG00000004864:ENSG00000092964

DEG.down	Brain_Amygdala	7678	46	5.01E-08	2.70E-06	ENSG00000021826:ENSG00000070371:ENSG00000070010:ENSG00000215012:ENSG00000183597:ENSG00000128191:ENSG00000099899:ENSG00000099904:ENSG00000099917:ENSG00000114302:ENSG00000178537:ENSG00000177479:ENSG00000178252:ENSG00000178149:ENSG00000198218:ENSG00000172037:ENSG00000177352:ENSG00000185909:ENSG00000188315:ENSG00000114316:ENSG00000145020:ENSG00000173402:ENSG00000164062:ENSG00000173531:ENSG00000164068:ENSG00000173540:ENSG00000185614:ENSG00000182179:ENSG00000183763:ENSG00000004534:ENSG00000003756:ENSG00000001617:ENSG00000214706:ENSG00000243477:ENSG00000114378:ENSG00000068001:ENSG00000007402:ENSG00000114735:ENSG00000114738:ENSG00000196455:ENSG00000034533:ENSG00000114686:ENSG00000138246:ENSG00000240303:ENSG00000113971:ENSG00000004864
DEG.down	Brain_Putamen_basal_ganglia	7776	46	7.54E-08	4.07E-06	ENSG00000070371:ENSG00000070010:ENSG00000215012:ENSG00000099889:ENSG00000183597:ENSG00000128191:ENSG00000099899:ENSG00000099901:ENSG00000099904:ENSG00000234409:ENSG00000099917:ENSG00000114302:ENSG00000178537:ENSG00000177479:ENSG00000178252:ENSG00000178149:ENSG00000198218:ENSG00000172037:ENSG00000177352:ENSG00000185909:ENSG00000188315:ENSG00000114316:ENSG00000145020:ENSG00000145029:ENSG00000173402:ENSG00000164062:ENSG00000173531:ENSG00000164068:ENSG00000173540:ENSG00000185614:ENSG00000182179:ENSG00000004534:ENSG00000003756:ENSG00000001617:ENSG00000214706:ENSG00000114378:ENSG00000068001:ENSG00000114735:ENSG00000114738:ENSG00000196455:ENSG00000034533:ENSG00000114686:ENSG00000138246:ENSG00000240303:ENSG00000113971:ENSG00000004864
DEG.twoside	Brain_Hippocampus	9458	51	1.69E-07	9.11E-06	ENSG00000021826:ENSG00000070371:ENSG00000070010:ENSG00000215012:ENSG00000099889:ENSG00000183597:ENSG00000128191:ENSG00000099899:ENSG00000099901:ENSG00000099904:ENSG00000234409:ENSG00000099917:ENSG00000114302:ENSG00000178537:ENSG00000177479:ENSG00000178252:ENSG00000178149:ENSG00000198218:ENSG00000172037:ENSG00000177352:ENSG00000185909:ENSG00000188315:ENSG00000114316:ENSG00000145020:ENSG00000173402:ENSG00000164061:ENSG00000164062:ENSG00000173531:ENSG00000164068:ENSG00000173540:ENSG00000185614:ENSG00000182179:ENSG00000183763:ENSG00000164076:ENSG00000001617:ENSG00000214706:ENSG00000243477:ENSG00000114378:ENSG00000068001:ENSG00000007402:ENSG00000114735:ENSG00000114738:ENSG00000088538:ENSG00000196455:ENSG00000034533:ENSG00000114686:ENSG00000138246:ENSG00000240303:ENSG00000113971:ENSG00000124664:ENSG00000004864
DEG.down	Brain_Nucleus_accumbens_basal_ganglia	6409	39	7.99E-07	4.32E-05	ENSG00000070371:ENSG00000070010:ENSG00000215012:ENSG00000099889:ENSG00000183597:ENSG00000128191:ENSG00000099899:ENSG00000099901:ENSG00000099904:ENSG00000099917:ENSG00000114302:ENSG00000178537:ENSG00000177479:ENSG00000178149:ENSG00000198218:ENSG00000172037:ENSG00000177352:ENSG00000185909:ENSG00000188315:ENSG00000114316:ENSG00000145020:ENSG00000173402:ENSG00000173531:ENSG00000164068:ENSG00000173540:ENSG00000185614:ENSG00000182179:ENSG00000001617:ENSG00000214706:ENSG00000114378:ENSG00000068001:ENSG00000114735:ENSG00000114738:ENSG00000196455:ENSG00000034533:ENSG00000138246:ENSG00000240303:ENSG00000113971:ENSG00000004864
DEG.twoside	Heart_Left_Ventricle	9979	51	1.06E-06	5.74E-05	ENSG00000115365:ENSG00000100075:ENSG00000070371:ENSG00000100084:ENSG00000070010:ENSG00000215012:ENSG00000093010:ENSG00000099889:ENSG00000128191:ENSG00000099899:ENSG00000099901:ENSG00000099904:ENSG00000099917:ENSG00000213672:ENSG00000114302:ENSG00000178467:ENSG00000178252:ENSG00000178149:ENSG00000198218:ENSG00000177352:ENSG00000188315:ENSG00000114316:ENSG00000145022:ENSG00000145020:ENSG00000145029:ENSG00000173531:ENSG00000164068:ENSG00000173540:ENSG00000176095:ENSG00000185614:ENSG00000182179:ENSG00000164077:ENSG00000003756:ENSG00000001617:ENSG00000243477:ENSG00000114378:ENSG00000114383:ENSG00000114735:ENSG00000114738:ENSG00000088538:ENSG00000196455:ENSG00000172260:ENSG00000034533:ENSG00000114686:ENSG00000138246:ENSG00000240303:ENSG00000113971:ENSG00000129048:ENSG00000125388:ENSG00000004864:ENSG00000092964

DEG.down	Heart_Left_Ventricle	9443	49	1.41E-06	7.61E-05	ENSG00000115365:ENSG00000100075:ENSG00000070371:ENSG00000100084:ENSG00000070010:ENSG00000215012:ENSG00000093010:ENSG00000099889:ENSG00000128191:ENSG00000099899:ENSG00000099901:ENSG00000099904:ENSG00000099917:ENSG00000213672:ENSG00000114302:ENSG00000178467:ENSG00000178252:ENSG00000178149:ENSG00000198218:ENSG00000177352:ENSG00000188315:ENSG00000114316:ENSG00000145022:ENSG00000145020:ENSG00000145029:ENSG00000173531:ENSG00000164068:ENSG00000173540:ENSG00000176095:ENSG00000185614:ENSG00000182179:ENSG00000164077:ENSG00000003756:ENSG00000001617:ENSG00000243477:ENSG00000114383:ENSG00000114735:ENSG00000088538:ENSG00000196455:ENSG00000017260:ENSG00000034533:ENSG00000114686:ENSG00000138246:ENSG00000240303:ENSG00000113971:ENSG00000129048:ENSG00000125388:ENSG00000004864:ENSG00000092964
DEG.twoside	Brain_Nucleus_accumbens_basal_ganglia	8860	47	1.53E-06	8.26E-05	ENSG00000070371:ENSG00000070010:ENSG00000215012:ENSG00000099889:ENSG00000183597:ENSG00000128191:ENSG00000099899:ENSG00000099901:ENSG00000099904:ENSG00000099917:ENSG00000008300:ENSG00000114302:ENSG00000178537:ENSG00000177479:ENSG00000178467:ENSG00000178149:ENSG00000198218:ENSG00000172037:ENSG00000177352:ENSG00000185909:ENSG00000188315:ENSG00000114316:ENSG00000145020:ENSG00000173402:ENSG00000164061:ENSG00000173531:ENSG00000164068:ENSG00000173540:ENSG00000185614:ENSG00000182179:ENSG00000183763:ENSG00000164076:ENSG00000001617:ENSG00000214706:ENSG00000114378:ENSG00000068001:ENSG00000007402:ENSG00000114735:ENSG00000114738:ENSG00000088538:ENSG00000196455:ENSG00000034533:ENSG00000114670:ENSG00000138246:ENSG00000240303:ENSG00000113971:ENSG00000004864
DEG.down	Brain_Substantia_nigra	7162	41	1.79E-06	9.66E-05	ENSG00000070371:ENSG00000070010:ENSG00000215012:ENSG00000099889:ENSG00000128191:ENSG00000099899:ENSG00000099904:ENSG00000234409:ENSG00000099917:ENSG00000114302:ENSG00000178537:ENSG00000177479:ENSG00000178252:ENSG00000178149:ENSG00000198218:ENSG00000172037:ENSG00000177352:ENSG00000185909:ENSG00000188315:ENSG00000114316:ENSG00000145020:ENSG00000145029:ENSG00000173402:ENSG00000164062:ENSG00000173531:ENSG00000164068:ENSG00000173540:ENSG00000176095:ENSG00000185614:ENSG00000182179:ENSG00000001617:ENSG00000214706:ENSG00000243477:ENSG00000114378:ENSG00000068001:ENSG00000114735:ENSG00000114738:ENSG00000196455:ENSG00000034533:ENSG00000138246:ENSG00000113971
DEG.twoside	Whole_Blood	8941	47	2.02E-06	1.09E-04	ENSG00000115365:ENSG00000100075:ENSG00000070371:ENSG00000100084:ENSG00000215012:ENSG00000093010:ENSG00000099889:ENSG00000183597:ENSG00000128191:ENSG00000099899:ENSG00000099901:ENSG00000099904:ENSG00000213672:ENSG00000114302:ENSG00000177479:ENSG00000178467:ENSG00000178252:ENSG00000178149:ENSG00000198218:ENSG00000172037:ENSG00000177352:ENSG00000185909:ENSG00000188315:ENSG00000145020:ENSG00000145029:ENSG00000173402:ENSG00000164062:ENSG00000173531:ENSG00000173540:ENSG00000185614:ENSG00000164077:ENSG00000004534:ENSG00000214706:ENSG00000243477:ENSG00000068001:ENSG00000114383:ENSG00000007402:ENSG00000114735:ENSG00000196455:ENSG00000017260:ENSG00000034533:ENSG00000114686:ENSG00000138246:ENSG00000240303:ENSG00000113971:ENSG00000004864:ENSG00000092964
DEG.down	Brain_Spinal_cord_cervical_c-1	5083	33	2.26E-06	1.22E-04	ENSG00000070371:ENSG00000215012:ENSG00000099889:ENSG00000128191:ENSG00000099904:ENSG00000234409:ENSG00000099917:ENSG00000114302:ENSG00000178149:ENSG00000198218:ENSG00000172037:ENSG00000177352:ENSG00000185909:ENSG00000114316:ENSG00000145020:ENSG00000145029:ENSG00000173402:ENSG00000164062:ENSG00000173531:ENSG00000164068:ENSG00000173540:ENSG00000185614:ENSG00000182179:ENSG00000183763:ENSG00000001617:ENSG00000214706:ENSG00000186792:ENSG00000114378:ENSG00000068001:ENSG00000114735:ENSG00000114738:ENSG00000196455:ENSG00000138246

DEG.down	Pancreas	9586	49	2.28E-06	1.23E-04	ENSG00000115365:ENSG00000070371:ENSG00000100084:ENSG00000070010:ENSG00000215012:ENSG00000093010:ENSG00000099889:ENSG00000183597:ENSG00000128191:ENSG00000099899:ENSG00000099901:ENSG00000099904:ENSG00000099917:ENSG00000213672:ENSG00000114302:ENSG00000178537:ENSG00000177479:ENSG00000178057:ENSG00000198218:ENSG00000177352:ENSG00000185909:ENSG00000188315:ENSG00000114316:ENSG00000145022:ENSG00000145029:ENSG00000173531:ENSG00000164068:ENSG00000176095:ENSG00000182179:ENSG00000164077:ENSG00000001617:ENSG00000114353:ENSG00000186792:ENSG00000243477:ENSG00000114378:ENSG00000068001:ENSG00000114383:ENSG00000007402:ENSG00000196455:ENSG00000017260:ENSG00000034533:ENSG00000114670:ENSG00000198585:ENSG00000138246:ENSG00000240303:ENSG00000113971:ENSG00000125388:ENSG00000004864:ENSG00000092964
DEG.down	Brain_Caudate_basal_ganglia	6858	39	4.66E-06	2.51E-04	ENSG00000070371:ENSG00000070010:ENSG00000215012:ENSG00000099889:ENSG00000183597:ENSG00000128191:ENSG00000099899:ENSG00000099901:ENSG00000099904:ENSG00000234409:ENSG00000099917:ENSG00000114302:ENSG00000178537:ENSG00000177479:ENSG00000178149:ENSG00000198218:ENSG00000172037:ENSG00000177352:ENSG00000185909:ENSG00000145020:ENSG00000173402:ENSG00000164062:ENSG00000173531:ENSG00000164068:ENSG00000173540:ENSG00000185614:ENSG00000182179:ENSG00000001617:ENSG00000214706:ENSG00000114378:ENSG00000068001:ENSG00000114735:ENSG00000114738:ENSG00000196455:ENSG00000034533:ENSG00000138246:ENSG00000240303:ENSG00000113971:ENSG00000004864
DEG.twoside	Brain_Amygdala	9498	48	4.69E-06	2.53E-04	ENSG00000021826:ENSG00000070371:ENSG00000070010:ENSG00000215012:ENSG00000183597:ENSG00000128191:ENSG00000099899:ENSG00000099904:ENSG00000099917:ENSG00000114302:ENSG00000178537:ENSG00000177479:ENSG00000178252:ENSG00000178149:ENSG00000198218:ENSG00000172037:ENSG00000177352:ENSG00000185909:ENSG00000188315:ENSG00000114316:ENSG00000145020:ENSG00000173402:ENSG00000164061:ENSG00000164062:ENSG00000173531:ENSG00000164068:ENSG00000173540:ENSG00000185614:ENSG00000182179:ENSG00000183763:ENSG00000164076:ENSG00000004534:ENSG00000003756:ENSG00000001617:ENSG00000214706:ENSG00000243477:ENSG00000114378:ENSG00000068001:ENSG00000007402:ENSG00000114735:ENSG00000114738:ENSG00000196455:ENSG00000034533:ENSG00000114686:ENSG00000138246:ENSG00000240303:ENSG00000113971:ENSG00000004864
DEG.twoside	Brain_Putamen_basal_ganglia	9517	48	4.99E-06	2.69E-04	ENSG00000070371:ENSG00000070010:ENSG00000215012:ENSG00000099889:ENSG00000183597:ENSG00000128191:ENSG00000099899:ENSG00000099901:ENSG00000099904:ENSG00000234409:ENSG00000099917:ENSG00000114302:ENSG00000178537:ENSG00000177479:ENSG00000178252:ENSG00000178149:ENSG00000198218:ENSG00000172037:ENSG00000177352:ENSG00000185909:ENSG00000188315:ENSG00000114316:ENSG00000145020:ENSG00000145029:ENSG00000173402:ENSG00000164061:ENSG00000164062:ENSG00000173531:ENSG00000164068:ENSG00000173540:ENSG00000185614:ENSG00000182179:ENSG00000164076:ENSG00000004534:ENSG00000003756:ENSG00000001617:ENSG00000214706:ENSG00000114378:ENSG00000068001:ENSG00000114735:ENSG00000114738:ENSG00000196455:ENSG00000034533:ENSG00000114686:ENSG00000138246:ENSG00000240303:ENSG00000113971:ENSG00000004864
DEG.down	Brain_Anterior_cingulate_cortex_BA24	6651	38	6.05E-06	3.27E-04	ENSG00000021826:ENSG00000070371:ENSG00000070010:ENSG00000128191:ENSG00000099899:ENSG00000234409:ENSG00000099917:ENSG00000114302:ENSG00000178537:ENSG00000177479:ENSG00000178149:ENSG00000198218:ENSG00000172037:ENSG00000177352:ENSG00000185909:ENSG00000188315:ENSG00000114316:ENSG00000145020:ENSG00000173402:ENSG00000173531:ENSG00000164068:ENSG00000173540:ENSG00000185614:ENSG00000182179:ENSG00000183763:ENSG00000001617:ENSG00000214706:ENSG00000114378:ENSG00000068001:ENSG00000114735:ENSG00000114738:ENSG00000196455:ENSG00000034533:ENSG00000138246:ENSG00000240303:ENSG00000113971:ENSG00000124664:ENSG00000004864

DEG.twoside	Pancreas	10331	49	2.30E-05	1.24E-03	ENSG00000115365:ENSG00000070371:ENSG00000100084:ENSG00000070010:ENSG00000215012:ENSG00000093010:ENSG00000099889:ENSG00000183597:ENSG00000128191:ENSG00000099899:ENSG00000099901:ENSG00000099904:ENSG00000099917:ENSG00000213672:ENSG00000114302:ENSG00000178537:ENSG00000177479:ENSG00000178057:ENSG00000198218:ENSG00000177352:ENSG00000185909:ENSG00000188315:ENSG00000114316:ENSG00000145022:ENSG00000145029:ENSG00000173531:ENSG00000164068:ENSG00000176095:ENSG00000182179:ENSG00000164077:ENSG00000001617:ENSG00000114353:ENSG00000186792:ENSG00000243477:ENSG00000114378:ENSG00000068001:ENSG00000114383:ENSG00000007402:ENSG00000196455:ENSG00000017260:ENSG00000034533:ENSG00000114670:ENSG00000198585:ENSG00000138246:ENSG00000240303:ENSG00000113971:ENSG00000125388:ENSG00000004864:ENSG00000092964
DEG.down	Heart_Atrial_Appendage	8409	42	4.59E-05	2.48E-03	ENSG00000115365:ENSG00000021826:ENSG00000100075:ENSG00000100084:ENSG00000070010:ENSG00000215012:ENSG00000093010:ENSG00000099889:ENSG00000128191:ENSG00000099899:ENSG00000099901:ENSG00000099904:ENSG00000099917:ENSG00000114302:ENSG00000178467:ENSG00000178149:ENSG00000198218:ENSG00000177352:ENSG00000188315:ENSG00000114316:ENSG00000145022:ENSG00000145029:ENSG00000173531:ENSG00000164068:ENSG00000173540:ENSG00000176095:ENSG00000185614:ENSG00000182179:ENSG00000164077:ENSG00000001617:ENSG00000186792:ENSG00000243477:ENSG00000114735:ENSG00000088538:ENSG00000196455:ENSG00000017260:ENSG00000034533:ENSG00000114670:ENSG00000198585:ENSG00000138246:ENSG00000113971:ENSG00000004864
DEG.twoside	Brain_Caudate_basal_ganglia	9070	44	5.41E-05	2.92E-03	ENSG00000070371:ENSG00000070010:ENSG00000215012:ENSG00000099889:ENSG00000183597:ENSG00000128191:ENSG00000099899:ENSG00000099901:ENSG00000099904:ENSG00000234409:ENSG00000099917:ENSG00000114302:ENSG00000178537:ENSG00000177479:ENSG00000178149:ENSG00000198218:ENSG00000172037:ENSG00000177352:ENSG00000185909:ENSG00000145020:ENSG00000173402:ENSG00000164061:ENSG00000164062:ENSG00000173531:ENSG00000164068:ENSG00000173540:ENSG00000185614:ENSG00000182179:ENSG00000164076:ENSG00000001617:ENSG00000214706:ENSG00000114378:ENSG00000068001:ENSG00000007402:ENSG00000114735:ENSG00000114738:ENSG00000088538:ENSG00000196455:ENSG00000034533:ENSG00000114670:ENSG00000138246:ENSG00000240303:ENSG00000113971:ENSG00000004864
DEG.twoside	Brain_Spinal_cord_cervical_c-1	7307	38	5.74E-05	3.10E-03	ENSG00000115365:ENSG00000021826:ENSG00000070371:ENSG00000215012:ENSG00000099889:ENSG00000128191:ENSG00000099904:ENSG00000234409:ENSG00000099917:ENSG00000114302:ENSG00000178149:ENSG00000198218:ENSG00000172037:ENSG00000177352:ENSG00000185909:ENSG00000114316:ENSG00000145020:ENSG00000145029:ENSG00000173402:ENSG00000164062:ENSG00000173531:ENSG00000164068:ENSG00000173540:ENSG00000185614:ENSG00000182179:ENSG00000183763:ENSG00000001617:ENSG00000214706:ENSG00000186792:ENSG00000114378:ENSG00000068001:ENSG00000114735:ENSG00000114738:ENSG00000088538:ENSG00000196455:ENSG00000198585:ENSG00000138246:ENSG00000004864
DEG.twoside	Brain_Substantia_nigra	8807	43	6.11E-05	3.30E-03	ENSG00000021826:ENSG00000070371:ENSG00000070010:ENSG00000215012:ENSG00000099889:ENSG00000128191:ENSG00000099899:ENSG00000099904:ENSG00000234409:ENSG00000099917:ENSG00000114302:ENSG00000178537:ENSG00000177479:ENSG00000178252:ENSG00000178149:ENSG00000198218:ENSG00000172037:ENSG00000177352:ENSG00000185909:ENSG00000188315:ENSG00000114316:ENSG00000145020:ENSG00000145029:ENSG00000173402:ENSG00000164062:ENSG00000173531:ENSG00000164068:ENSG00000173540:ENSG00000176095:ENSG00000185614:ENSG00000182179:ENSG00000001617:ENSG00000214706:ENSG00000243477:ENSG00000114378:ENSG00000068001:ENSG00000114735:ENSG00000114738:ENSG00000196455:ENSG00000034533:ENSG00000198585:ENSG00000138246:ENSG00000113971
DEG.twoside	Heart_Atrial_Appendage	9152	44	6.85E-05	3.70E-03	ENSG00000115365:ENSG00000021826:ENSG00000100075:ENSG00000100084:ENSG00000070010:ENSG00000215012:ENSG00000093010:ENSG00000099889:ENSG00000128191:ENSG00000099899:ENSG00000099901:ENSG00000099904:ENSG00000099917:ENSG00000114302:ENSG00000178467:ENSG00000178149:ENSG00000198218:ENSG00000177352:ENSG00000188315:ENSG00000114316:ENSG00000145022:ENSG00000145029:ENSG00000173531:ENSG00000164068:ENSG00000173540:ENSG00000176095:ENSG00000185614:ENSG00000182179:ENSG00000164077:ENSG00000001617:ENSG00000186792:ENSG000

						00243477:ENSG00000007402:ENSG00000114735:ENSG00000114738:ENSG00000088538:ENSG00000196455:ENSG00000017260:ENSG00000034533:ENSG00000114670:ENSG00000198585:ENSG00000138246:ENSG00000113971:ENSG00000004864
DEG.down	Brain_Frontal_Cortex_BA9	5015	29	0.00011	5.96E-03	ENSG00000021826:ENSG00000070371:ENSG00000070010:ENSG00000128191:ENSG00000114302:ENSG00000178537:ENSG00000177479:ENSG00000198218:ENSG00000172037:ENSG00000177352:ENSG00000185909:ENSG00000145020:ENSG00000173402:ENSG00000173531:ENSG00000173540:ENSG00000185614:ENSG00000182179:ENSG00000183763:ENSG00000001617:ENSG00000214706:ENSG00000114378:ENSG00000068001:ENSG00000114735:ENSG00000114738:ENSG00000138246:ENSG00000240303:ENSG00000113971:ENSG00000124664:ENSG00000004864
DEG.down	Brain_Cerebellar_Hemisphere	2607	19	0.00014	7.34E-03	ENSG00000183597:ENSG00000178537:ENSG00000172037:ENSG00000177352:ENSG00000185909:ENSG00000173402:ENSG00000173540:ENSG00000182179:ENSG00000164078:ENSG00000001617:ENSG00000214706:ENSG00000114378:ENSG00000068001:ENSG00000114735:ENSG00000114738:ENSG00000114670:ENSG00000113971:ENSG00000124664:ENSG00000004864
DEG.twoside	Brain_Anterior_cingulate_cortex_BA24	9021	42	0.00026	1.39E-02	ENSG00000021826:ENSG00000070371:ENSG00000070010:ENSG00000128191:ENSG00000099899:ENSG00000234409:ENSG00000099917:ENSG00000008300:ENSG00000114302:ENSG00000178537:ENSG00000177479:ENSG00000178149:ENSG00000198218:ENSG00000172037:ENSG00000177352:ENSG00000185909:ENSG00000188315:ENSG00000114316:ENSG00000145020:ENSG00000173402:ENSG00000164061:ENSG00000173531:ENSG00000164068:ENSG00000173540:ENSG00000185614:ENSG00000182179:ENSG00000183763:ENSG00000164076:ENSG00000001617:ENSG00000214706:ENSG00000114378:ENSG00000068001:ENSG00000114735:ENSG00000114738:ENSG00000088538:ENSG00000196455:ENSG00000034533:ENSG00000138246:ENSG00000240303:ENSG00000113971:ENSG00000124664:ENSG00000004864
DEG.down	Brain_Hypothalamus	5904	31	0.00035	1.89E-02	ENSG00000070371:ENSG00000215012:ENSG00000128191:ENSG00000099899:ENSG00000099917:ENSG00000114302:ENSG00000178537:ENSG00000177479:ENSG00000178149:ENSG00000198218:ENSG00000172037:ENSG00000177352:ENSG00000114316:ENSG00000145020:ENSG00000173402:ENSG00000173531:ENSG00000164068:ENSG00000173540:ENSG00000185614:ENSG00000182179:ENSG00000001617:ENSG00000214706:ENSG00000114378:ENSG00000068001:ENSG00000114735:ENSG00000114738:ENSG00000196455:ENSG00000138246:ENSG00000240303:ENSG00000113971:ENSG00000004864
DEG.down	Colon_Transverse	1583	13	0.0006	3.24E-02	ENSG00000021826:ENSG00000070371:ENSG00000184058:ENSG00000234409:ENSG00000008300:ENSG00000173531:ENSG00000185614:ENSG00000007402:ENSG00000114738:ENSG00000088538:ENSG00000114670:ENSG00000129048:ENSG00000125388
DEG.twoside	Brain_Frontal_Cortex_BA9	8147	37	0.00133	7.18E-02	ENSG00000021826:ENSG00000070371:ENSG00000070010:ENSG00000128191:ENSG00000008300:ENSG00000213672:ENSG00000114302:ENSG00000178537:ENSG00000177479:ENSG00000178467:ENSG00000198218:ENSG00000172037:ENSG00000177352:ENSG00000185909:ENSG00000145020:ENSG00000173402:ENSG00000164061:ENSG00000173531:ENSG00000173540:ENSG00000185614:ENSG00000182179:ENSG00000183763:ENSG00000164076:ENSG00000164077:ENSG00000001617:ENSG00000214706:ENSG00000186792:ENSG00000114378:ENSG00000068001:ENSG00000114735:ENSG00000114738:ENSG00000088538:ENSG00000138246:ENSG00000240303:ENSG00000113971:ENSG00000124664:ENSG00000004864
DEG.down	Brain_Cortex	5556	28	0.00148	8.01E-02	ENSG00000021826:ENSG00000070371:ENSG00000070010:ENSG00000128191:ENSG00000114302:ENSG00000178537:ENSG00000177479:ENSG00000198218:ENSG00000172037:ENSG00000177352:ENSG00000185909:ENSG00000145020:ENSG00000173402:ENSG00000173531:ENSG00000173540:ENSG00000185614:ENSG00000182179:ENSG00000001617:ENSG00000214706:ENSG00000114378:ENSG00000068001:ENSG00000114735:ENSG00000114738:ENSG00000138246:ENSG00000240303:ENSG00000113971:ENSG00000124664:ENSG00000004864

DEG.twoside	Liver	9510	41	0.00177	9.57E-02	ENSG00000115365:ENSG00000021826:ENSG00000070371:ENSG00000070010:ENSG00000215012:ENSG00000099889:ENSG00000183597:ENSG00000128191:ENSG00000099904:ENSG00000099917:ENSG00000213672:ENSG00000114302:ENSG00000178537:ENSG00000177479:ENSG00000178467:ENSG00000178149:ENSG00000198218:ENSG00000177352:ENSG00000185909:ENSG00000188315:ENSG00000114316:ENSG00000145029:ENSG00000173402:ENSG00000173531:ENSG00000176095:ENSG00000182179:ENSG00000003756:ENSG00000001617:ENSG00000243477:ENSG00000114378:ENSG00000068001:ENSG00000114383:ENSG00000114738:ENSG00000196455:ENSG00000017260:ENSG00000034533:ENSG00000138246:ENSG00000240303:ENSG00000113971:ENSG00000004864:ENSG00000092964
DEG.twoside	Brain_Cerebellum	8903	39	0.00185	9.97E-02	ENSG00000115365:ENSG00000100084:ENSG00000215012:ENSG00000099889:ENSG00000183597:ENSG00000128191:ENSG00000099899:ENSG00000099904:ENSG00000234409:ENSG00000008300:ENSG00000213672:ENSG00000114302:ENSG00000178537:ENSG00000178467:ENSG00000172037:ENSG00000177352:ENSG00000185909:ENSG00000188315:ENSG00000145020:ENSG00000145029:ENSG00000164061:ENSG00000164068:ENSG00000173540:ENSG00000176095:ENSG00000182179:ENSG00000164078:ENSG00000001617:ENSG00000186792:ENSG00000243477:ENSG00000114378:ENSG00000068001:ENSG00000007402:ENSG00000114735:ENSG00000114738:ENSG00000088538:ENSG00000113971:ENSG00000125388:ENSG00000124664:ENSG00000004864
DEG.down	Brain_Cerebellum	2739	17	0.00196	1.06E-01	ENSG00000183597:ENSG00000114302:ENSG00000178537:ENSG00000172037:ENSG00000177352:ENSG00000185909:ENSG00000173540:ENSG00000182179:ENSG00000164078:ENSG00000001617:ENSG00000114378:ENSG00000068001:ENSG00000114735:ENSG00000114738:ENSG00000113971:ENSG00000124664:ENSG00000004864
DEG.twoside	Brain_Hypothalamus	8435	37	0.00259	1.40E-01	ENSG00000070371:ENSG00000215012:ENSG00000128191:ENSG00000099899:ENSG00000099917:ENSG00000008300:ENSG00000114302:ENSG00000178537:ENSG00000177479:ENSG00000178149:ENSG00000198218:ENSG00000172037:ENSG00000177352:ENSG00000114316:ENSG00000145020:ENSG00000173402:ENSG00000164061:ENSG00000173531:ENSG00000164068:ENSG00000173540:ENSG00000185614:ENSG00000182179:ENSG00000164076:ENSG00000001617:ENSG00000214706:ENSG00000114378:ENSG00000068001:ENSG00000007402:ENSG00000114735:ENSG00000114738:ENSG00000088538:ENSG00000196455:ENSG00000114670:ENSG00000138246:ENSG00000240303:ENSG00000113971:ENSG00000004864
DEG.down	Cells_EBV-transformed_lymphocytes	2383	15	0.00325	1.76E-01	ENSG00000021826:ENSG00000099904:ENSG00000178467:ENSG00000185909:ENSG00000188315:ENSG00000145020:ENSG00000173402:ENSG00000164068:ENSG00000185614:ENSG00000164078:ENSG00000068001:ENSG00000198585:ENSG00000113971:ENSG00000125388:ENSG00000092964
DEG.twoside	Brain_Cerebellar_Hemisphere	8908	38	0.00368	1.98E-01	ENSG00000115365:ENSG00000100084:ENSG00000215012:ENSG00000099889:ENSG00000183597:ENSG00000128191:ENSG00000099904:ENSG00000008300:ENSG00000213672:ENSG00000178537:ENSG00000178467:ENSG00000172037:ENSG00000177352:ENSG00000185909:ENSG00000188315:ENSG00000145020:ENSG00000145029:ENSG00000173402:ENSG00000164061:ENSG00000164068:ENSG00000173540:ENSG00000176095:ENSG00000182179:ENSG00000164078:ENSG00000001617:ENSG00000214706:ENSG00000186792:ENSG00000114378:ENSG00000068001:ENSG00000007402:ENSG00000114735:ENSG00000114738:ENSG00000088538:ENSG00000114670:ENSG00000113971:ENSG00000125388:ENSG00000124664:ENSG00000004864
DEG.down	Liver	7985	35	0.00371	2.00E-01	ENSG00000115365:ENSG00000070371:ENSG00000070010:ENSG00000215012:ENSG00000099889:ENSG00000183597:ENSG00000128191:ENSG00000099904:ENSG00000099917:ENSG00000213672:ENSG00000114302:ENSG00000177479:ENSG00000178467:ENSG00000178149:ENSG00000198218:ENSG00000177352:ENSG00000185909:ENSG00000188315:ENSG00000114316:ENSG00000145029:ENSG00000173402:ENSG00000176095:ENSG00000182179:ENSG00000003756:ENSG00000001617:ENSG00000243477:ENSG00000068001:ENSG00000114383:ENSG00000114738:ENSG00000196455:ENSG00000017260:ENSG00000034533:ENSG00000138246:ENSG00000113971:ENSG00000092964

DEG.twoside	Brain_Cortex	8348	36	0.00418	2.26E-01	ENSG00000021826:ENSG00000070371:ENSG00000070010:ENSG00000128191:ENSG00000008300:ENSG00000213672:ENSG00000114302:ENSG00000178537:ENSG00000177479:ENSG00000178467:ENSG00000198218:ENSG00000172037:ENSG00000177352:ENSG00000185909:ENSG00000145020:ENSG00000173402:ENSG00000164061:ENSG00000173531:ENSG00000173540:ENSG00000185614:ENSG00000182179:ENSG00000164076:ENSG00000164077:ENSG00000001617:ENSG00000214706:ENSG00000186792:ENSG00000114378:ENSG00000068001:ENSG00000114735:ENSG00000114738:ENSG00000088538:ENSG00000138246:ENSG00000240303:ENSG00000113971:ENSG00000124664:ENSG00000004864
DEG.up	Thyroid	5687	26	0.00883	4.77E-01	ENSG00000100084:ENSG00000184058:ENSG00000215012:ENSG00000099889:ENSG00000183597:ENSG00000128191:ENSG00000099899:ENSG00000099904:ENSG00000178467:ENSG00000178149:ENSG00000177352:ENSG00000145022:ENSG00000145020:ENSG00000145029:ENSG00000173531:ENSG00000173540:ENSG00000182179:ENSG00000001617:ENSG00000068001:ENSG00000114735:ENSG00000114738:ENSG00000088538:ENSG00000196455:ENSG00000034533:ENSG00000114670:ENSG00000113971
DEG.down	Ovary	1521	10	0.01221	6.59E-01	ENSG00000183597:ENSG00000234409:ENSG00000008300:ENSG00000178537:ENSG00000185614:ENSG00000164078:ENSG00000001617:ENSG00000114378:ENSG00000088538:ENSG00000129048
DEG.down	Kidney_Cortex	5544	25	0.01233	6.66E-01	ENSG00000115365:ENSG00000021826:ENSG00000070371:ENSG00000070010:ENSG00000215012:ENSG00000128191:ENSG00000114302:ENSG00000177479:ENSG00000198218:ENSG00000177352:ENSG00000185909:ENSG00000188315:ENSG00000114316:ENSG00000176095:ENSG00000185614:ENSG00000183763:ENSG00000186792:ENSG00000243477:ENSG00000196455:ENSG00000017260:ENSG00000034533:ENSG00000138246:ENSG00000113971:ENSG00000125388:ENSG00000092964
DEG.down	Stomach	2607	14	0.01698	9.17E-01	ENSG00000115365:ENSG00000070371:ENSG00000184058:ENSG00000234409:ENSG00000008300:ENSG00000213672:ENSG00000188315:ENSG00000114316:ENSG00000173531:ENSG00000185614:ENSG00000088538:ENSG00000114670:ENSG00000125388:ENSG00000092964
DEG.twoside	Thyroid	6725	28	0.02184	1.00E+00	ENSG00000100084:ENSG00000184058:ENSG00000215012:ENSG00000099889:ENSG00000183597:ENSG00000128191:ENSG00000099899:ENSG00000099904:ENSG00000008300:ENSG00000178467:ENSG00000178149:ENSG00000177352:ENSG00000145022:ENSG00000145020:ENSG00000145029:ENSG00000173531:ENSG00000173540:ENSG00000185614:ENSG00000182179:ENSG00000001617:ENSG00000068001:ENSG00000114735:ENSG00000114738:ENSG00000088538:ENSG00000196455:ENSG00000034533:ENSG00000114670:ENSG00000113971
DEG.twoside	Nerve_Tibial	6774	28	0.0239	1.00E+00	ENSG00000070371:ENSG00000099889:ENSG00000099899:ENSG00000099904:ENSG00000234409:ENSG00000213672:ENSG00000178467:ENSG00000178149:ENSG00000177352:ENSG00000145020:ENSG00000145029:ENSG00000173531:ENSG00000182179:ENSG00000164078:ENSG00000186792:ENSG00000243477:ENSG00000068001:ENSG00000007402:ENSG00000114738:ENSG00000088538:ENSG00000196455:ENSG00000017260:ENSG00000034533:ENSG00000198585:ENSG00000138246:ENSG00000240303:ENSG00000113971:ENSG00000125388
DEG.down	Esophagus_Mucosa	3580	17	0.02606	1.00E+00	ENSG00000070371:ENSG00000099889:ENSG00000183597:ENSG00000213672:ENSG00000178537:ENSG00000178467:ENSG00000145022:ENSG00000145020:ENSG00000145029:ENSG00000173531:ENSG00000114378:ENSG00000088538:ENSG00000114670:ENSG00000198585:ENSG00000113971:ENSG00000125388:ENSG00000092964
DEG.twoside	Artery_Aorta	4216	19	0.03038	1.00E+00	ENSG00000070371:ENSG00000184058:ENSG00000099889:ENSG00000183597:ENSG00000234409:ENSG00000213672:ENSG00000145020:ENSG00000145029:ENSG00000173531:ENSG00000185614:ENSG00000182179:ENSG00000001617:ENSG00000114378:ENSG00000114738:ENSG00000088538:ENSG00000138246:ENSG00000240303:ENSG00000113971:ENSG00000129048
DEG.down	Spleen	2069	11	0.0356	1.00E+00	ENSG00000021826:ENSG00000070371:ENSG00000184058:ENSG00000114302:ENSG00000173402:ENSG00000164078:ENSG00000007402:ENSG00000088538:ENSG00000114670:ENSG00000129048:ENSG00000125388
DEG.twoside	Colon_Transverse	3185	15	0.03859	1.00E+00	ENSG00000021826:ENSG00000070371:ENSG00000184058:ENSG00000234409:ENSG00000008300:ENSG00000173531:ENSG00000185614:ENSG00000164078:ENSG00000007402:ENSG00000114738:ENSG00000088538:ENSG00000114670:ENSG00000129048:ENSG00000125388:ENSG00000124664
DEG.down	Vagina	647	5	0.03941	1.00E+00	ENSG00000021826:ENSG00000008300:ENSG00000007402:ENSG00000088538:ENSG00000129048

DEG.up	Nerve_Tibial	5869	24	0.04205	1.00E+00	ENSG00000070371:ENSG00000099889:ENSG00000099899:ENSG00000099904:ENSG00000234409:ENSG00000213672:ENSG00000178467:ENSG00000178149:ENSG00000177352:ENSG00000145020:ENSG00000145029:ENSG00000173531:ENSG00000182179:ENSG00000243477:ENSG00000068001:ENSG00000088538:ENSG00000196455:ENSG00000017260:ENSG00000034533:ENSG00000198585:ENSG00000138246:ENSG00000240303:ENSG00000113971:ENSG00000125388
DEG.up	Muscle_Skeletal	1143	7	0.0472	1.00E+00	ENSG00000070371:ENSG00000184058:ENSG00000114302:ENSG00000178537:ENSG00000177479:ENSG00000164068:ENSG00000114738
DEG.twoside	Kidney_Cortex	6871	27	0.04779	1.00E+00	ENSG00000115365:ENSG00000021826:ENSG00000070371:ENSG00000070010:ENSG00000215012:ENSG00000128191:ENSG00000114302:ENSG00000177479:ENSG00000198218:ENSG00000177352:ENSG00000185909:ENSG00000188315:ENSG00000114316:ENSG00000173531:ENSG00000176095:ENSG00000185614:ENSG00000183763:ENSG00000186792:ENSG00000243477:ENSG00000114378:ENSG00000196455:ENSG00000017260:ENSG00000034533:ENSG00000138246:ENSG00000113971:ENSG00000125388:ENSG00000092964
Enriched gene-sets at nominal p-value <0.05 were reported.						

Supplemental table S8. Differential gene set enrichment in 30 general tissue types from GTEx.

Category	GeneSet	N_genes	N_overlap	p	adjP	genes
DEG.twoside	Muscle	7979	51	3.27E-10	9.80E-09	ENSG00000115365:ENSG00000100075:ENSG00000070371:ENSG00000184058:ENSG00000215012:ENSG00000093010:ENSG00000099889:ENSG00000128191:ENSG00000099899:ENSG00000099901:ENSG00000099904:ENSG00000213672:ENSG00000114302:ENSG00000178537:ENSG00000177479:ENSG00000178467:ENSG00000178149:ENSG00000198218:ENSG00000177352:ENSG00000188315:ENSG00000114316:ENSG00000145022:ENSG00000145020:ENSG00000145029:ENSG00000173531:ENSG00000164068:ENSG00000173540:ENSG00000176095:ENSG00000185614:ENSG00000182179:ENSG00000183763:ENSG00000004534:ENSG00000001617:ENSG00000114353:ENSG00000243477:ENSG00000114378:ENSG00000068001:ENSG00000007402:ENSG00000114735:ENSG00000114738:ENSG00000088538:ENSG00000196455:ENSG00000017260:ENSG00000034533:ENSG00000198585:ENSG00000138246:ENSG00000240303:ENSG00000113971:ENSG00000125388:ENSG00000004864:ENSG00000092964
DEG.down	Muscle	6836	44	1.39E-08	4.17E-07	ENSG00000115365:ENSG00000100075:ENSG00000215012:ENSG00000093010:ENSG00000099889:ENSG00000128191:ENSG00000099899:ENSG00000099901:ENSG00000099904:ENSG00000213672:ENSG00000178467:ENSG00000178149:ENSG00000198218:ENSG00000177352:ENSG00000188315:ENSG00000114316:ENSG00000145022:ENSG00000145020:ENSG00000145029:ENSG00000173531:ENSG00000173540:ENSG00000176095:ENSG00000185614:ENSG00000182179:ENSG00000183763:ENSG00000004534:ENSG00000001617:ENSG00000114353:ENSG00000243477:ENSG00000114378:ENSG00000068001:ENSG00000007402:ENSG00000114735:ENSG00000088538:ENSG00000196455:ENSG00000017260:ENSG00000034533:ENSG00000198585:ENSG00000138246:ENSG00000240303:ENSG00000113971:ENSG00000125388:ENSG00000004864:ENSG00000092964
DEG.down	Pancreas	9586	49	2.28E-06	6.84E-05	ENSG00000115365:ENSG00000070371:ENSG00000100084:ENSG00000070010:ENSG00000215012:ENSG00000093010:ENSG00000099889:ENSG00000183597:ENSG00000128191:ENSG00000099899:ENSG00000099901:ENSG00000099904:ENSG00000099917:ENSG00000213672:ENSG00000114302:ENSG00000178537:ENSG00000177479:ENSG00000178057:ENSG00000198218:ENSG00000177352:ENSG00000185909:ENSG00000188315:ENSG00000114316:ENSG00000145022:ENSG00000145029:ENSG00000173531:ENSG00000164068:ENSG00000176095:ENSG00000182179:ENSG00000164077:ENSG00000001617:ENSG00000114353:ENSG00000186792:ENSG00000243477:ENSG00000114378:ENSG00000068001:ENSG00000114383:ENSG00000007402:ENSG00000196455:ENSG00000017260:ENSG00000034533:ENSG00000114670:ENSG00000198585:ENSG00000138246:ENSG00000240303:ENSG00000113971:ENSG00000125388:ENSG00000004864:ENSG00000092964

DEG.down	Blood	6184	37	2.86E-06	8.58E-05	ENSG00000115365:ENSG00000100075:ENSG00000070371:ENSG00000215012:ENSG00000093010:ENSG00000099889:ENSG00000128191:ENSG00000099901:ENSG00000099904:ENSG00000213672:ENSG00000114302:ENSG00000177479:ENSG00000178467:ENSG00000178252:ENSG00000178149:ENSG00000198218:ENSG00000172037:ENSG00000185909:ENSG00000145020:ENSG00000145029:ENSG00000173402:ENSG00000173531:ENSG00000185614:ENSG00000164077:ENSG00000214706:ENSG00000243477:ENSG00000068001:ENSG00000007402:ENSG00000114735:ENSG00000196455:ENSG00000017260:ENSG00000114686:ENSG00000138246:ENSG00000240303:ENSG00000113971:ENSG00000004864:ENSG00000092964
DEG.twoside	Pancreas	10331	49	2.30E-05	0.00069032	ENSG00000115365:ENSG00000070371:ENSG00000100084:ENSG00000070010:ENSG00000215012:ENSG00000093010:ENSG00000099889:ENSG00000183597:ENSG00000128191:ENSG00000099899:ENSG00000099901:ENSG00000099904:ENSG00000099917:ENSG00000213672:ENSG00000114302:ENSG00000178537:ENSG00000177479:ENSG00000178057:ENSG00000198218:ENSG00000177352:ENSG00000185909:ENSG00000188315:ENSG00000114316:ENSG00000145022:ENSG00000145029:ENSG00000173531:ENSG00000164068:ENSG00000176095:ENSG00000182179:ENSG00000164077:ENSG00000001617:ENSG00000114353:ENSG00000186792:ENSG00000243477:ENSG00000114378:ENSG00000068001:ENSG00000114383:ENSG00000007402:ENSG00000196455:ENSG00000017260:ENSG00000034533:ENSG00000114670:ENSG00000198585:ENSG00000138246:ENSG00000240303:ENSG00000113971:ENSG00000125388:ENSG00000004864:ENSG00000092964
DEG.twoside	Heart	9960	46	0.00011453	0.00343591	ENSG00000115365:ENSG00000100075:ENSG00000100084:ENSG00000070010:ENSG00000215012:ENSG00000093010:ENSG00000099889:ENSG00000128191:ENSG00000099899:ENSG00000099901:ENSG00000099904:ENSG00000099917:ENSG00000114302:ENSG00000178467:ENSG00000178149:ENSG00000198218:ENSG00000177352:ENSG00000188315:ENSG00000114316:ENSG00000145022:ENSG00000145029:ENSG00000173531:ENSG00000164068:ENSG00000173540:ENSG00000176095:ENSG00000185614:ENSG00000182179:ENSG00000164077:ENSG00000001617:ENSG00000186792:ENSG00000243477:ENSG00000114378:ENSG00000114383:ENSG00000114735:ENSG00000114738:ENSG00000088538:ENSG00000196455:ENSG00000017260:ENSG00000034533:ENSG00000114670:ENSG00000198585:ENSG00000138246:ENSG00000240303:ENSG00000113971:ENSG00000125388:ENSG00000004864
DEG.down	Heart	9347	44	0.000118	0.0035401	ENSG00000115365:ENSG00000100075:ENSG00000100084:ENSG00000070010:ENSG00000215012:ENSG00000093010:ENSG00000099889:ENSG00000128191:ENSG00000099899:ENSG00000099901:ENSG00000099904:ENSG00000099917:ENSG00000114302:ENSG00000178467:ENSG00000178149:ENSG00000198218:ENSG00000177352:ENSG00000188315:ENSG00000114316:ENSG00000145022:ENSG00000145029:ENSG00000173531:ENSG00000164068:ENSG00000173540:ENSG00000176095:ENSG00000185614:ENSG00000182179:ENSG00000164077:ENSG00000001617:ENSG00000186792:ENSG00000243477:ENSG00000114383:ENSG00000114735:ENSG00000088538:ENSG00000196455:ENSG00000017260:ENSG00000034533:ENSG00000114670:ENSG00000198585:ENSG00000138246:ENSG00000240303:ENSG00000113971:ENSG00000125388:ENSG00000004864
DEG.twoside	Blood	8434	40	0.00027332	0.00819957	ENSG00000115365:ENSG00000100075:ENSG00000070371:ENSG00000093009:ENSG00000215012:ENSG00000093010:ENSG00000099889:ENSG00000183597:ENSG00000128191:ENSG00000099901:ENSG00000099904:ENSG00000213672:ENSG00000114302:ENSG00000177479:ENSG00000178467:ENSG00000178252:ENSG00000178149:ENSG00000198218:ENSG00000172037:ENSG00000185909:ENSG00000188315:ENSG00000145020:ENSG00000145029:ENSG00000173402:ENSG00000173531:ENSG00000185614:ENSG00000164077:ENSG00000214706:ENSG00000243477:ENSG00000068001:ENSG00000007402:ENSG00000114735:ENSG00000196455:ENSG00000017260:ENSG00000114686:ENSG00000138246:ENSG00000240303:ENSG00000113971:ENSG00000004864:ENSG00000092964
DEG.down	Brain	5503	29	0.00056083	0.01682482	ENSG00000070371:ENSG00000070010:ENSG00000183597:ENSG00000099917:ENSG00000114302:ENSG00000178537:ENSG00000177479:ENSG00000178149:ENSG00000198218:ENSG00000172037:ENSG00000177352:ENSG00000185909:ENSG00000173402:ENSG00000164062:ENSG00000173531:ENSG00000173540:ENSG00000185614:ENSG00000182179:ENSG00000001617:ENSG00000214706:ENSG00000114378:ENSG00000068001:ENSG00000114735:ENSG00000114738:ENSG00000196455:ENSG00000138246:ENSG00000240303:ENSG00000113971:ENSG00000004864

DEG.twoside	Liver	9510	41	0.00177281	0.05318443	ENSG00000115365:ENSG00000021826:ENSG00000070371:ENSG00000070010:ENSG00000215012:ENSG00000099889:ENSG00000183597:ENSG00000128191:ENSG00000099904:ENSG00000099917:ENSG00000213672:ENSG00000114302:ENSG00000178537:ENSG00000177479:ENSG00000178467:ENSG00000178149:ENSG00000198218:ENSG00000177352:ENSG00000185909:ENSG00000188315:ENSG00000114316:ENSG00000145029:ENSG00000173402:ENSG00000173531:ENSG00000176095:ENSG00000182179:ENSG00000003756:ENSG00000001617:ENSG00000243477:ENSG00000114378:ENSG00000068001:ENSG00000114383:ENSG00000114738:ENSG00000196455:ENSG00000017260:ENSG00000034533:ENSG00000138246:ENSG00000240303:ENSG00000113971:ENSG00000004864:ENSG00000092964
DEG.twoside	Brain	8711	38	0.00240413	0.07212392	ENSG00000115365:ENSG00000070371:ENSG00000070010:ENSG00000183597:ENSG00000099917:ENSG00000008300:ENSG00000114302:ENSG00000178537:ENSG00000177479:ENSG00000178467:ENSG00000178149:ENSG00000198218:ENSG00000172037:ENSG00000177352:ENSG00000185909:ENSG00000173402:ENSG00000164061:ENSG00000164062:ENSG00000173531:ENSG00000173540:ENSG00000185614:ENSG00000182179:ENSG00000164076:ENSG00000001617:ENSG00000214706:ENSG00000186792:ENSG00000114378:ENSG00000068001:ENSG00000007402:ENSG00000114735:ENSG00000114738:ENSG00000088538:ENSG00000196455:ENSG00000138246:ENSG00000240303:ENSG00000113971:ENSG00000004864:ENSG00000092964
DEG.down	Liver	7985	35	0.00370878	0.11126355	ENSG00000115365:ENSG00000070371:ENSG00000070010:ENSG00000215012:ENSG00000099889:ENSG00000183597:ENSG00000128191:ENSG00000099904:ENSG00000099917:ENSG00000213672:ENSG00000114302:ENSG00000177479:ENSG00000178467:ENSG00000178149:ENSG00000198218:ENSG00000177352:ENSG00000185909:ENSG00000188315:ENSG00000114316:ENSG00000145029:ENSG00000173402:ENSG00000176095:ENSG00000182179:ENSG00000003756:ENSG00000001617:ENSG00000243477:ENSG00000068001:ENSG00000114383:ENSG00000114738:ENSG00000196455:ENSG00000017260:ENSG00000034533:ENSG00000138246:ENSG00000113971:ENSG00000092964
DEG.down	Colon	1378	10	0.00633741	0.19012222	ENSG00000021826:ENSG00000070371:ENSG00000184058:ENSG00000234409:ENSG00000173531:ENSG00000185614:ENSG00000114378:ENSG00000007402:ENSG00000114738:ENSG00000129048
DEG.up	Thyroid	5687	26	0.00882731	0.2648194	ENSG00000100084:ENSG00000184058:ENSG00000215012:ENSG00000099889:ENSG00000183597:ENSG00000128191:ENSG00000099899:ENSG00000099904:ENSG00000178467:ENSG00000178149:ENSG00000177352:ENSG00000145022:ENSG00000145020:ENSG00000145029:ENSG00000173531:ENSG00000173540:ENSG00000182179:ENSG00000001617:ENSG00000068001:ENSG00000114735:ENSG00000114738:ENSG00000088538:ENSG00000196455:ENSG00000034533:ENSG00000114670:ENSG00000113971
DEG.down	Ovary	1521	10	0.01220929	0.36627884	ENSG00000183597:ENSG00000234409:ENSG00000008300:ENSG00000178537:ENSG00000185614:ENSG00000164078:ENSG00000001617:ENSG00000114378:ENSG00000088538:ENSG00000129048
DEG.up	Skin	3125	16	0.01636585	0.49097563	ENSG00000021826:ENSG00000093009:ENSG00000184058:ENSG00000185838:ENSG00000215012:ENSG00000178149:ENSG00000177352:ENSG00000185614:ENSG00000183763:ENSG00000164078:ENSG00000001617:ENSG00000114378:ENSG00000114738:ENSG00000017260:ENSG00000129048:ENSG00000124664
DEG.down	Stomach	2607	14	0.01698304	0.50949123	ENSG00000115365:ENSG00000070371:ENSG00000184058:ENSG00000234409:ENSG00000008300:ENSG00000213672:ENSG00000188315:ENSG00000114316:ENSG00000173531:ENSG00000185614:ENSG00000088538:ENSG00000114670:ENSG00000125388:ENSG00000092964
DEG.twoside	Thyroid	6725	28	0.02184192	0.6552575	ENSG00000100084:ENSG00000184058:ENSG00000215012:ENSG00000099889:ENSG00000183597:ENSG00000128191:ENSG00000099899:ENSG00000099904:ENSG00000008300:ENSG00000178467:ENSG00000178149:ENSG00000177352:ENSG00000145022:ENSG00000145020:ENSG00000145029:ENSG00000173531:ENSG00000173540:ENSG00000185614:ENSG00000182179:ENSG00000001617:ENSG00000068001:ENSG00000114735:ENSG00000114738:ENSG00000088538:ENSG00000196455:ENSG00000034533:ENSG00000114670:ENSG00000113971

DEG.twoside	Nerve	6774	28	0.02390169	0.71705068	ENSG00000070371:ENSG00000099889:ENSG00000099899:ENSG00000099904:ENSG00000234409:ENSG00000213672:ENSG00000178467:ENSG00000178149:ENSG00000177352:ENSG00000145020:ENSG00000145029:ENSG00000173531:ENSG00000182179:ENSG00000164078:ENSG00000186792:ENSG00000243477:ENSG00000068001:ENSG00000007402:ENSG00000114738:ENSG00000088538:ENSG00000196455:ENSG00000017260:ENSG00000034533:ENSG00000198585:ENSG00000138246:ENSG00000240303:ENSG00000113971:ENSG00000125388
DEG.down	Kidney	5412	23	0.03167529	0.95025857	ENSG00000115365:ENSG00000021826:ENSG00000070371:ENSG00000070010:ENSG00000215012:ENSG00000128191:ENSG00000114302:ENSG00000177479:ENSG00000198218:ENSG00000177352:ENSG00000188315:ENSG00000114316:ENSG00000185614:ENSG00000183763:ENSG00000186792:ENSG00000243477:ENSG00000196455:ENSG00000017260:ENSG00000034533:ENSG00000138246:ENSG00000113971:ENSG00000125388:ENSG00000092964
DEG.down	Spleen	2069	11	0.03559843	1	ENSG00000021826:ENSG00000070371:ENSG00000184058:ENSG00000114302:ENSG00000173402:ENSG00000164078:ENSG00000007402:ENSG00000088538:ENSG00000114670:ENSG00000129048:ENSG00000125388
DEG.down	Vagina	647	5	0.03941349	1	ENSG00000021826:ENSG00000008300:ENSG00000007402:ENSG00000088538:ENSG00000129048
DEG.twoside	Skin	5256	22	0.04144981	1	ENSG00000021826:ENSG00000093009:ENSG00000184058:ENSG00000185838:ENSG00000215012:ENSG00000099889:ENSG00000008300:ENSG00000178467:ENSG00000178149:ENSG00000177352:ENSG00000185614:ENSG00000183763:ENSG00000164078:ENSG00000001617:ENSG00000114378:ENSG00000007402:ENSG00000114738:ENSG00000088538:ENSG00000017260:ENSG00000129048:ENSG00000125388:ENSG00000124664
DEG.up	Nerve	5869	24	0.04204588	1	ENSG00000070371:ENSG00000099889:ENSG00000099899:ENSG00000099904:ENSG00000234409:ENSG00000213672:ENSG00000178467:ENSG00000178149:ENSG00000177352:ENSG00000145020:ENSG00000145029:ENSG00000173531:ENSG00000182179:ENSG00000243477:ENSG00000068001:ENSG00000088538:ENSG00000196455:ENSG00000017260:ENSG00000034533:ENSG00000198585:ENSG00000138246:ENSG00000240303:ENSG00000113971:ENSG00000125388
DEG.up	Muscle	1143	7	0.04719654	1	ENSG00000070371:ENSG00000184058:ENSG00000114302:ENSG00000178537:ENSG00000177479:ENSG00000164068:ENSG00000114738
Enriched gene-sets at nominal p-value <0.05 were reported.						

Supplemental table S9. Colocalization of *RNF123* locus with muscle skeletal eQTL signals.

Locus		CWP GWAS				Muscle eQTL				LD	PP	
GWAS	eQTL	Independent SNP	MAF	N	P	Lead eSNP	MAF	N	P	r^2	PP3	PP4
RNF123	*CDHR4*	rs1491985	0.18	249843	1.6E-08	rs6809879	019	706	3.1E-08	1	0.07	0.93

CWP, chronic widespread musculoskeletal pain; DRG, dorsal root ganglion; eQTL, expression quantitative trait loci; MAF, minor allele frequency; N, sample size; P, p-value; LD, linkage disequilibrium; r^2, the pairwise LD between the independent GWAS SNP, and the lead eSNP; PP, posterior probability; PP3, the posterior probabilities for having separate variants for both traits, PP4, the posterior probabilities for having shared SNP between two traits. eQTL SNP with lowest p-value was reported.

Supplemental table S10. Colocalization of *RNF123* locus with DRG eQTL signals at exon-level.

Locus		CWP GWAS				DRG eQTL				LD	PP	
GWAS	eQTL	Independent SNP	MAF	N	P	Lead eSNP	MAF	N	P	r^2	PP3	PP4
RNF123	*APEH*	rs1491985	0.18	249843	3.40E-08	rs13093525	0.16	214	1.32E-06	1	0.01	0.72

CWP, chronic widespread musculoskeletal pain; DRG, dorsal root ganglion; eQTL, expression quantitative trait loci; MAF, minor allele frequency; N, sample size; P, p-value; LD, linkage disequilibrium; r^2, the pairwise LD between the independent GWAS SNP, and the lead eSNP; PP, posterior probability; PP3, the posterior probabilities for having separate variants for both traits, PP4, the posterior probabilities for having shared SNP between two traits. eQTL SNP with lowest p-value was reported.

REFERENCES

1. Fry A, Littlejohns TJ, Sudlow C, Doherty N, Adamska L, Sprosen T, et al. Comparison of Sociodemographic and Health-Related Characteristics of UK Biobank Participants With Those of the General Population. Am J Epidemiol. 2017 Nov 1; 186(9):1026-1034.

2. Bycroft C, Freeman C, Petkova D, Band G, Elliott LT, Sharp K, et al. The UK Biobank resource with deep phenotyping and genomic data. Nature. 2018 2018/10/01; 562(7726):203-209.

3. Hagen K, Linde M, Heuch I, Stovner LJ, Zwart JA. Increasing prevalence of chronic musculoskeletal complaints. A large 11-year follow-up in the general population (HUNT 2 and 3). Pain Med. 2011 Nov; 12(11):1657-1666.

4. Krokstad S, Langhammer A, Hveem K, Holmen T, Midthjell K, Stene T, et al. Cohort Profile: The HUNT Study, Norway. International Journal of Epidemiology. 2012; 42(4):968-977.

5. Moayyeri A, Hammond CJ, Valdes AM, Spector TD. Cohort Profile: TwinsUK and healthy ageing twin study. Int J Epidemiol. 2013 Feb; 42(1):76-85.

6. Ikram MA, Brusselle GGO, Murad SD, van Duijn CM, Franco OH, Goedegebure A, et al. The Rotterdam Study: 2018 update on objectives, design and main results. Eur J Epidemiol. 2017 Sep; 32(9):807-850.

7. Peters MJ, Broer L, Willemen HL, Eiriksdottir G, Hocking LJ, Holliday KL, et al. Genome-wide association study meta-analysis of chronic widespread pain: evidence for involvement of the 5p15.2 region. Ann Rheum Dis. 2013 Mar; 72(3):427-436.

8. Steptoe A, Breeze E, Banks J, Nazroo J. Cohort Profile: The English Longitudinal Study of Ageing. Int J Epidemiol. 2013; 42(6):1640-1648.

9. Uhlig BL, Sand T, Nilsen TI, Mork PJ, Hagen K. Insomnia and risk of chronic musculoskeletal complaints: longitudinal data from the HUNT study, Norway. BMC Musculoskelet Disord. 2018 Apr 25; 19(1):128.

10. Kuorinka I, Jonsson B, Kilbom A, Vinterberg H, Biering-Sorensen F, Andersson G, et al. Standardised Nordic questionnaires for the analysis of musculoskeletal symptoms. Appl Ergon. 1987 Sep; 18(3):233-237.

11. Franzblau A, Salerno DF, Armstrong TJ, Werner RA. Test-retest reliability of an upper-extremity discomfort questionnaire in an industrial population. Scand J Work Environ Health. 1997 Aug; 23(4):299-307.

12. White KP, Harth M, Speechley M, Ostbye T. Testing an instrument to screen for fibromyalgia syndrome in general population studies: the London Fibromyalgia Epidemiology Study Screening Questionnaire. J Rheumatol. 1999 Apr; 26(4):880-884.

13. Purcell S, Neale B, Todd-Brown K, Thomas L, Ferreira MA, Bender D, et al. PLINK: a tool set for whole-genome association and population-based linkage analyses. Am J Hum Genet. 2007 Sep; 81(3):559-575.

14. Loh P-R, Tucker G, Bulik-Sullivan BK, Vilhjálmsson BJ, Finucane HK, Salem RM, et al. Efficient Bayesian mixed-model analysis increases association power in large cohorts. Nature Genetics. 2015 2015/03/01; 47(3):284-290.

15. Yang J, Ferreira T, Morris AP, Medland SE, Madden PAF, Heath AC, et al. Conditional and joint multiple-SNP analysis of GWAS summary statistics identifies additional variants influencing complex traits. Nature Genetics. 2012 2012/04/01; 44(4):369-375.

16. Yang J, Lee SH, Goddard ME, Visscher PM. GCTA: a tool for genome-wide complex trait analysis. Am J Hum Genet. 2011 Jan 7; 88(1):76-82.

17. Watanabe K, Taskesen E, van Bochoven A, Posthuma D. Functional mapping and annotation of genetic associations with FUMA. Nature Communications. 2017 2017/11/28; 8(1):1826.

18. Zhou W, Nielsen JB, Fritsche LG, Dey R, Gabrielsen ME, Wolford BN, et al. Efficiently controlling for case-control imbalance and sample relatedness in large-scale genetic association studies. Nat Genet. 2018 Sep; 50(9):1335-1341.

19. Zhou X, Stephens M. Efficient multivariate linear mixed model algorithms for genome-wide association studies. Nat Methods. 2014 Apr; 11(4):407-409.

20. Willer CJ, Li Y, Abecasis GR. METAL: fast and efficient meta-analysis of genomewide association scans. *Bioinformatics*; 2010:2190-2191.

21. Bulik-Sullivan BK, Loh P-R, Finucane HK, Ripke S, Yang J, Patterson N, et al. LD Score regression distinguishes confounding from polygenicity in genome-wide association studies. Nature Genetics. 2015 2015/03/01; 47(3):291-295.

22. Loh P-R, Bhatia G, Gusev A, Finucane HK, Bulik-Sullivan BK, Pollack SJ, et al. Contrasting genetic architectures of schizophrenia and other complex diseases using fast variance-components analysis. Nature genetics. 2015; 47(12):1385-1392.

23. Zheng J, Erzurumluoglu AM, Elsworth BL, Kemp JP, Howe L, Haycock PC, et al. LD Hub: a centralized database and web interface to perform LD score regression that maximizes the potential of summary level GWAS data for SNP heritability and genetic correlation analysis. Bioinformatics. 2016; 33(2):272-279.

24. Wang K, Li M, Hakonarson H. ANNOVAR: functional annotation of genetic variants from high-throughput sequencing data. Nucleic Acids Res. 2010; 38(16):e164.

25. de Leeuw CA, Mooij JM, Heskes T, Posthuma D. MAGMA: Generalized Gene-Set Analysis of GWAS Data. PLOS Computational Biology. 2015; 11(4):e1004219.

26. Ramasamy A, Trabzuni D, Guelfi S, Varghese V, Smith C, Walker R, et al. Genetic variability in the regulation of gene expression in ten regions of the human brain. Nature Neuroscience. 2014 2014/10/01; 17(10):1418-1428.

27. Parisien M, Khoury S, Chabot-Doré A-J, Sotocinal SG, Slade GD, Smith SB, et al. Effect of Human Genetic Variability on Gene Expression in Dorsal Root Ganglia and Association with Pain Phenotypes. Cell Reports. 2017; 19(9):1940-1952.

28. Consortium TG. The GTEx Consortium atlas of genetic regulatory effects across human tissues. Science. 2020; 369(6509):1318.

29. Giambartolomei C, Vukcevic D, Schadt EE, Franke L, Hingorani AD, Wallace C, et al. Bayesian test for colocalisation between pairs of genetic association studies using summary statistics. PLoS Genet. 2014 May; 10(5):e1004383.

30. Allen N, Sudlow C, Downey P, Peakman T, Danesh J, Elliott P, et al. UK Biobank: Current status and what it means for epidemiology. Health Policy and Technology. 2012 2012/09/01/; 1(3):123-126.

31. Anderson CD, Nalls MA, Biffi A, Rost NS, Greenberg SM, Singleton AB, et al. The effect of survival bias on case-control genetic association studies of highly lethal diseases. Circ Cardiovasc Genet. 2011 Apr; 4(2):188-196.

32. Chanock SJ, Manolio T, Boehnke M, Boerwinkle E, Hunter DJ, Thomas G, et al. Replicating genotype-phenotype associations. Nature. 2007 Jun 7; 447(7145):655-660.
